# Simple modeling of familial Alzheimer’s disease using human pluripotent stem cell-derived cerebral organoid technology

**DOI:** 10.1186/s13287-024-03732-1

**Published:** 2024-04-24

**Authors:** Mu Seog Choe, Han Cheol Yeo, Joong Sun Kim, Jean Lee, Hae Jun Lee, Hyung-Ryong Kim, Kyung Min Baek, Na-Yeon Jung, Murim Choi, Min Young Lee

**Affiliations:** 1 Department of Molecular Physiology, College of Pharmacy, Research Institute of Pharmaceutical Sciences, Vessel-Organ Interaction Research Center (VOICE, MRC), Kyungpook National University, 41566 Daegu, Republic of Korea; 2 Department of Veterinary Anatomy, College of Veterinary Medicine, Chonnam National University, 61186 Gwangju, Republic of Korea; 3 Department of Biochemistry and Biomedical Sciences, College of Medicine, Seoul National University, 03080 Seoul, Republic of Korea; 4 Division of Radiation Biomedical Research, Korea Institute of Radiological & Medical Sciences (KIRAMS), 01812 Seoul, Republic of Korea; 5 Department of Pharmacology, College of Dentistry, Jeonbuk National University, 54896 Jeonju, Republic of Korea; 6 Department of Cardiovascular and Neurologic Disease, College of Oriental Medicine, Daegu Haany University, 42158 Daegu, Republic of Korea; 7 Department of Neurology, Pusan National University Yangsan Hospital, Pusan National University School of Medicine, 50612 Yangsan, Republic of Korea

**Keywords:** Cerebral organoids, Human pluripotent stem cells, Alzheimer’s disease, Disease modeling, Drug screening

## Abstract

**Background:**

Cerebral organoids (COs) are the most advanced in vitro models that resemble the human brain. The use of COs as a model for Alzheimer’s disease (AD), as well as other brain diseases, has recently gained attention. This study aimed to develop a human AD CO model using normal human pluripotent stem cells (hPSCs) that recapitulates the pathological phenotypes of AD and to determine the usefulness of this model for drug screening.

**Methods:**

We established AD hPSC lines from normal hPSCs by introducing genes that harbor familial AD mutations, and the COs were generated using these hPSC lines. The pathological features of AD, including extensive amyloid-β (Aβ) accumulation, tauopathy, and neurodegeneration, were analyzed using enzyme-linked immunosorbent assay, Amylo-Glo staining, thioflavin-S staining, immunohistochemistry, Bielschowsky’s staining, and western blot analysis.

**Results:**

The AD COs exhibited extensive Aβ accumulation. The levels of paired helical filament tau and neurofibrillary tangle-like silver deposits were highly increased in the AD COs. The number of cells immunoreactive for cleaved caspase-3 was significantly increased in the AD COs. In addition, treatment of AD COs with BACE1 inhibitor IV, a β-secretase inhibitor, and compound E, a γ-secretase inhibitor, significantly attenuated the AD pathological features.

**Conclusion:**

Our model effectively recapitulates AD pathology. Hence, it is a valuable platform for understanding the mechanisms underlying AD pathogenesis and can be used to test the efficacy of anti-AD drugs.

**Supplementary Information:**

The online version contains supplementary material available at 10.1186/s13287-024-03732-1.

## Background

Alzheimer’s disease (AD) is the most common form of dementia. Its pathological features include the excessive accumulation of amyloid β (Aβ) peptides, and the formation of neurofibrillary tangles (NFTs), which are composed of hyperphosphorylated tau protein, and neurodegeneration [1, 2]. To date, various models have been developed to study AD. Animal models, including those carrying the familial AD (fAD) mutations, have been developed to mimic human AD pathology, and these models have contributed tremendously to the understanding of this disease. However, these models are unable to fully recapitulate all of the disease features because of interspecies differences between humans and animals [3]. To overcome these limitations of animal models, human AD modeling technologies using human neural cell systems have been developed. However, 2D neural cell culture systems poorly represent the central nervous system (CNS) environment because they typically exclude the complex 3D architecture and cellular composition of the brain, although they are likely to capture the early-stage pathology, such as neuronal degeneration by exogenous Aβ [4]. In addition, inducing the typical AD pathology, such as Aβ accumulation or NFTs, poses a challenge in 2D models. Ravi et al. argued that 3D cell culture conditions were advantageous over 2D conditions because of their ability to provide spatial structuring, adhesion, proliferation, signaling, and mechanical cell transduction, which 2D cells cannot provide [5, 6]. Recently, Choi et al. successfully reproduced AD pathology, including the deposition of Aβ and accumulation of phosphorylated tau protein, in a Matrigel matrix-based 3D neural progenitor cell culture system [7]. However, this model did not recapitulate the histological structure and cellular composition of the human brain in vivo.

*Human pluripotent stem cells (hPSCs), including human embryonic stem cells (hESCs) and human-induced pluripotent stem cells (hiPSCs), have unique features, including self-renewal and pluripotency. Recent advancements in differentiation techniques using hPSCs have led to the development of organoid technology, which mimics the organ’s structural and functional features.* Since the development of hPSC-derived cerebral organoid (CO) technology, efforts to apply this technology to the modeling of neurological disorders and their related therapeutic discoveries are increasingly being made. COs are an advanced form of in vitro models that are capable of resembling the human brain in the CO formation process, cellular or structural composition, and intercellular connections. COs have great potential to create qualified models of CNS diseases, including AD, and provide a platform to validate the disease mechanisms [8–10]. Many researchers have discussed their expectations regarding the potential of using CO technology to model AD as well as other diseases of the brain [3, 11].

*AD can be broadly categorized into two main types based on its occurrence: late-onset or sporadic (sAD) and early-onset or fAD. sAD accounts for the majority (> 95%) of AD cases and typically manifests after the age of 65 years. On the other hand, fAD is less common but tends to occur at an earlier age. Although fAD represents a smaller proportion of cases, most recent drug development efforts, including the first FDA-approved drug, Aducanumab, are based on understanding the mechanisms that underlie fAD onset* [12]. In this study, a human AD modeling strategy was developed using a normal hPSC line *based on fAD*. We began this study with the simple hypothesis that if an AD hPSC line is established by the transduction of *f*AD genes into a normal hPSC line, an indefinite number of AD COs can be generated from the transgenic AD hPSC lines, and the AD COs can be used as a 3D in vitro human AD model. *There is no need to perform multiple transduction experiments as a stable AD hPSC line can be generated due to the characteristic self-renewal of hPSCs.* As expected, our AD CO model showed robust expression of AD pathologies, including Aβ accumulation and tauopathy-paired helical filament (PHF)-tau expression, as well as the formation of NFT-like structures and increased cellular apoptosis. The amyloid/tau/neurodegeneration (ATN) framework has been proposed to represent the biological state of AD [13]. The results of this study indicate that we successfully recapitulated the key pathological phenotypes of AD in our AD CO model. As a reliable disease model, organoids can be used not only to model the disease and elucidate human pathophysiologies but also for the screening of potential therapeutic drugs. In this study, we demonstrated the usefulness of our AD CO model as a tool for drug screening. The AD CO modeling strategy presented here can be applied to the development of other brain disease models and may be extended to various diseases.

## Methods

### hPSC culture

The hESC line (SNUhES31) was obtained from the Institute of Reproductive Medicine and Population, Medical Research Center, Seoul National University Hospital, South Korea. *The hiPSC lines from a patient with early-onset AD (56 years old, female) were kindly provided by Dr. Na-Yeon Jung (Department of Neurology, Pusan National University Yangsan Hospital, Pusan National University School of Medicine, Republic of Korea).* The cells were cultured on 10 µg/mL mitomycin C-treated STO cells (ATCC) in hPSC medium (1% MEM-NEAA (Thermo Fisher Scientific), 1% GlutaMAX™ (Thermo Fisher Scientific), 7 µL/L β-mercaptoethanol, and 20% knockout serum replacement in DMEM/F-12) containing 20 ng/mL of basic fibroblast growth factor (bFGF). For feeder-free culture, the cells were cultured in Essential-8 medium (Thermo Fisher Scientific) on Geltrex^TM^-coated (Thermo Fisher Scientific) culture plates. The hPSCs were *subcultured* every four days using 500 µM EDTA-PBS.

### Establishment of AD hPSC lines

We constructed the lentiviral plasmid vectors *CAG-MCS, PGK-MCS, EF1α-MCS*, and *CMV-MCS* by editing the *pCAG-CreERT2*, *pSico-PGK-Puro*, *pCDH-EF1α-MCS-IRES-RFP* (System Bioscience), and *pLV-mCherry* plasmids. *pCAG-CreERT2* was gifted by Connie Cepko (Addgene, #14,797). The *pSico-PGK-puro* plasmid was gifted by Tyler Jacks (Addgene, #17,797), and *pLV-mCherry* was gifted by Pantelis Tsoulfas (Addgene, #36,084). Next, we generated *CAG-mCherry* (CmC), *PGK-mCherry* (PmC), *EF1α-mCherry* (EmC), and *CMV-mCherry* (CMmC) by inserting mCherry into multiple cloning sites. The constructs encoding the full-length amyloid precursor protein (*APP*) with the K670M/N671L (Swedish), I716V (Florida), and V717I (London) mutations (*APP*^*SweFlLon*^) and presenilin-1 (*PSEN1*) with the M146L and L286V mutations (*PSEN1*^*M146L/L286V*^) were cloned from the brain tissue of 8-month-old 5XFAD mice (B6SJL-Tg (APPSwFlLon, PSEN1*M146L*L286V)6799Vas/Mmjax; The Jackson Laboratory;http://www.jax.org/strain/006554*). The cloned sequences of APP and PSEN1 harboring the fAD mutations from 5XFAD mice are of human origin.* Total RNA was extracted using an RNA Extraction Kit (Qiagen) and reverse transcribed using a cDNA synthesis kit (Bio-Rad). *APP*^*SweFlLon*^ and *PSEN1*^*M146L/L286V*^ cDNA were PCR-amplified with restriction enzymes using KOD-Plus-Neo (Toyobo). The primers used for cloning were: APP-BamH I-F, 5′-GAATGGATCCATGCTGCCCGGTTTGGCACTG-3′ and APP-Mlu I-R, 5′-GAATACGCGTCTAGTTCTGCATCTGCTCAAA-3′; PSEN1-BamH I-F, GAATGGATCCATGACAGAGTTACCTGCACCG-3′ and PSEN1-Mlu I-R, GAATACGCGTCTAGATATAAAATTGATGGAA-3′. Next, *APP*^SweFlLon^ and *APP*^SweFlLon^*-IRES-PSEN1*^M146L/L286V^, which were constructed using the amplified *APP*^SweFlLon^ and *PSEN1*^M146L/L286V^ genes, were inserted into multiple cloning sites to obtain two additional constructs: *CAG-APP*^*SweFlLon*^ (CA) and *CAG-APP*^*SweFlLon*^-*IRES*- *PSEN1*^M146L/L286V^ (CAP). All newly constructed vectors were confirmed by sequencing (Enzynomics).

CA or CAP lentiviral particles were produced by cotransfection of each plasmid with the packaging plasmids (an envelope plasmid harboring *VSVg* gene and another plasmid harboring the *gag-pol* genes) into 85% confluent HEK293T cells (ATCC) in a 10-mm tissue culture plate using the FuGENE® HD transfection reagent (Roche). The medium was changed at 24 h after transfection, and 10 mL of virus-containing medium was harvested once a day for three days. Each aliquot of the collected medium (30 mL) was concentrated to 200 µL by ultracentrifugation (25,000 rpm) at 4 °C for 2 h (Hitachi). Next, feeder-free cultured hPSCs (hESCs or hiPSCs) in a well of the 12-well tissue culture plate were transfected with CA or CAP viral particles by changing the medium to Essential-8 medium containing 2 × 10^7^ IU/mL concentrated viral particles and 2 µg/mL hexadimethrine bromide (Sigma-Aldrich). The medium was changed every day for three days. Five hundred hPSCs transfected with CA or CAP viral particles were plated into the STO-plated well of a 6-well plate (Corning). After 7 days of culture, every cell-derived colony was manually dissected and transferred into the wells of a Matrigel-coated 12-well culture plate. After expansion under feeder-free conditions, the expression levels of APP and PSEN1 (CTF) were analyzed by western blotting.

The hESCs transfected with the CA or CAP lentiviral particles were named the CA hESC line and CAP hESC line, respectively, and the hiPSCs transfected with the CAP lentiviral particles were named the CAP hiPSC line. Each cell line was imaged using an inverted microscope in bright-field or fluorescence mode (DM IL LED Fluo; Leica).

#### Whole-genome sequencing

*Whole-genome sequencing of the CAP hESC line was performed using the Illumina NovaSeq 6000 at a mean coverage depth of 30×. BWA-mem v.0.7.17* [14] *was used with the default options to map the raw reads of 151 bp to the human reference genome (GRCh38) with the CAP lentiviral vector sequence. The resulting alignment file in the BAM format was sorted using Samtools v.1.16.1. Chimeric reads aligned to both vector and flanking human genome sequences allowed us to locate the vector integration site.*

#### Single-cell RNA sequencing (scRNA-seq)

*Raw reads from the scRNA-seq analysis were processed with Cell Ranger v.7.1.0 (10X Genomics) using the human reference genome (GRCh38) to generate the unique molecular identifier count matrices. Normalization and cell-type clustering were performed using the Seurat v.5.0.1 package in R (v.4.2.2)* [15]. *High-quality cells were selected based on the number of genes detected (200–2,500), and cells with high mitochondrial counts (> 10%) were excluded. The FindClusters function was used to identify cell clusters (resolution 0.65), and the cells were clustered and visualized using the UMAP method* [16].

### CO culture

COs were generated using a previously established protocol [17]. In brief, embryoid bodies (EBs) were formed by plating 9,000 feeder-free cultured hPSCs (hESCs or hiPSCs) dissociated into single cells into the wells of 96-well ultra-low-attachment plates (Corning) containing hPSC medium supplemented with 50 µM of Y27632, a ROCK inhibitor (Tocris), and 5 ng/mL of bFGF (day 0). The hPSC medium was replaced every other day for six days. On day 6, each EB was transferred to a 24-well ultra-low-attachment plate containing 500 µL of neural induction medium (1% MEM-NEAA, 1% GlutaMAX™, 1% N2 supplement (Thermo Fisher Scientific), and 1 µg/mL of heparin (Sigma-Aldrich) in DMEM/F-12). On day 8, 500 µL of fresh neural induction medium was added to each well. On day 10 of the protocol, the EBs were embedded in 20 µL droplets of Matrigel (BD Bioscience) and incubated in a gel for 1 h at 37 °C. These droplets were transferred and grown in CO differentiation medium without vitamin A (0.5% MEM-NEAA, 1% Glutamax, 1% B27 supplement without vitamin A, 0.5% N2 supplement, 2.5 µg/mL human insulin (Roche), and 3.5 µL/L β-mercaptoethanol in a 1:1 mixture of neurobasal medium (Thermo Fisher Scientific) and DMEM/F-12). The medium was changed on day 12, and on day 14, the Matrigel droplets were moved to a spinner flask containing CO differentiation medium (0.5% MEM-NEAA, 1% GlutaMAX™, B27 supplement (Thermo Fisher Scientific), 0.5% N2 supplement, 2.5 µg/mL human insulin, and 3.5 µL/L β-mercaptoethanol in a 1:1 mixture of neurobasal medium and DMEM/F-12). Thereafter, the medium was replaced every seven days.

### Neuronal differentiation

Single cell-dissociated hESCs (1 × 10^5^) were plated onto Matrigel-coated 24-well plates in mTeSR™1 supplemented with 5 µM of Y27632 (day − 4). On day − 3, the medium was replaced with mTeSR™1 without Y27632, and the medium was changed daily. On day 0, the mTeSR™1 medium was replaced with neuronal cell induction medium (1% MEM-NEAA, 1% GlutaMX™, 2% B27 supplement, 1% N2 supplement, and 20 µg/mL of human insulin in a 1:1 mixture of neurobasal medium and DMEM/F-12), and the medium was replaced every day until day 10. On day 11, the dissociated cells were resuspended in a neuronal cell induction medium supplemented with 5 µM of Y27632 and replated onto a Matrigel-coated 24-well plate (3 × 10^5^ cells/well). On day 13, the medium was changed to neuronal cell induction medium supplemented with 100 nM of LDN-193,189, 10 µM of SB431542, and 2 µM of XAV 939. The medium was refreshed every other day until day 17. On day 19, the medium was changed to a neuronal cell maturation medium (1% N2 supplement, 2% B-27 supplement, and 25 ng/mL of BDNF in neurobasal medium). The medium was then changed every other day. On day 25, the cells were dissociated and replated onto a Matrigel-coated 24-well plate (1 × 10^5^ cells/well), and the medium was replaced every other day.

### Immunocytochemistry

The dissociated cells were fixed with a 4% paraformaldehyde (PFA) solution in PBS (Wako). Cells were permeabilized and blocked with 10% normal goat serum (NGS) (Vector) in 0.1% PBST (vol/vol, Triton X-100 in PBS). The cells were incubated with primary antibodies in PBST containing 2% NGS, and the following primary antibodies were used against the proteins: Nanog (rabbit, Cell Signaling), Oct4 (mouse, Santa Cruz), Tra-1-60 (mouse, Santa Cruz), SSEA4 (mouse, Santa Cruz), and PHF-tau (mouse, BioLegend). After three washes with PBST, the cells were incubated with the goat antirabbit or goat antimouse Alexa Fluor™ 488 conjugated (Thermo Fisher Scientific) secondary antibodies. Images were obtained using confocal microscopy (Leica TCS SP5 II; Leica).

### Immunohistochemistry

The COs were fixed with a 4% PFA solution in PBS. The COs were allowed to sink in 15% and 30% sucrose solutions sequentially, followed by embedding in an optimal cutting temperature compound (Leica). The COs were then cryosctioned into 15-µm-thick slices using a cryotome (CM1850 cryostat; Leica). For immunohistochemistry, the sections were permeabilized and blocked with 10% NGS in PBST, and the sections were incubated with primary antibodies in PBST containing 2% NGS. Primary antibodies against the following proteins were used: 6E10 (mouse, BioLegend), 4G8 (mouse, BioLegend), PHF-tau (mouse, BioLegend), TUJ1 (rabbit, Cell Signaling), SOX2 (rabbit, Cell Signaling), TUJ1 (mouse, R&D Systems), TBR2 (mouse, R&D Systems), DCX (rabbit, Cell Signaling), PAX6 (rabbit, BioLegend), MAP2 (rabbit, Cell Signaling), MAP2 (mouse, Abcam), and N-cadherin (rabbit, Santa Cruz). After washing three times with PBST, the tissues were incubated with goat antirabbit or antimouse Alexa Fluor™ 488 and 647 conjugated (Thermo Fisher Scientific) secondary antibodies. Images were obtained using confocal microscopy (Leica TCS SP5 II; Leica).

### Amylo-Glo staining

Amylo-Glo staining was performed using the Amylo-Glo RTD® Amyloid Plaque Stain Reagent (Biosensis). Briefly, the cryosection slides were washed three times with distilled water (DW) and transferred to 70% ethanol at room temperature for 5 min. The slides were then rinsed in DW for 2 min without shaking. The slides were then incubated for 10 min in the prepared 1× staining solution and rinsed with PBS for 5 min without shaking. The slides were then briefly rinsed in fresh DW and mounted with coverslips using a mounting medium. Images were obtained using confocal microscopy (Leica TCS SP5 II; Leica).

### Thioflavin-S staining

The cryosection slides were washed three times with DW and incubated in 1% aqueous thioflavin-S (Sigma-Aldrich) solution (v/v in DW) for 15 min. The slides were then washed twice with 80% ethanol for 3 min before washing with 95% ethanol for 3 min. The slides were then briefly rinsed in fresh DW and mounted with coverslips using mounting medium. Images were obtained using confocal microscopy (Leica TCS SP5 II; Leica).

### Bielschowsky’s silver staining

Bielschowsky’s silver staining was performed using the VitroView™ Bielschowsky’s Silver Stain kit (VitroVivo Biotech). Briefly, the cryosectioned slides were incubated in a prewarmed (40 °C) silver nitrate solution for 15 min before rinsing with DW. The slides were incubated in ammonium silver solution at 40 °C for 30 min. The slides were placed in a Developer Stock Solution for 1 min, after which the reaction was halted by immersing the slides in a 1% ammonium hydroxide solution for 1 min. After three washes with DW, the slides were placed in a 5% sodium thiosulfate solution for 5 min before washing three times with DW. After dehydration with 100% ethanol, the slides were incubated in xylene, and the slides were then mounted with coverslips using a mounting medium. Images were obtained using a microscope (AXIO; Zeiss).

### Western blot analysis

The COs or neuronal cells were sonicated in RIPA buffer (iNtRON Biotechnology) on ice (Vibra-Cell™). The proteins that were obtained from each sample were separated using 10–12% SDS-PAGE resolving gels and transferred to polyvinylidene fluoride (PVDF) transfer membranes (Millipore). The membranes were washed with TBST (150 mM NaCl, 10 mM Tris-HCl [pH 7.6], and 0.1% Tween-20), blocked with 5% skimmed milk (Millipore) for 1 h, and incubated with primary antibodies. Primary antibodies against the following proteins were used: PHF-tau (mouse, BioLegend), Tau (mouse, Cell Signaling), amyloid precursor protein (APP) (mouse, Cell Signaling), presenilin (PSEN)-1 (rabbit, Cell Signaling), and β-actin (mouse, Santa Cruz). The membranes were washed with TBST and incubated with horseradish peroxidase (HRP)-linked goat antirabbit (Santa Cruz) or goat antimouse (Santa Cruz) IgG secondary antibodies. An enhanced chemiluminescence solution (Thermo Fisher Scientific) was used to visualize the bands. Images were obtained using the Chemidoc™ imaging system (Bio-Rad). Densitometric analysis was performed using ImageJ software (National Institutes of Health (NIH)).

### Enzyme-linked immunosorbent assay (ELISA)

The Aβ 1–40 and Aβ 1–42 levels were determined using commercial human amyloid-β assay kits (IBL). Samples were obtained from each CO sonicated in RIPA buffer on ice, and the sample proteins were quantified using the BCA method, and ELISA was performed using 10 µg of lysate, according to the manufacturer’s instructions. The absorbance was measured at 450 nm using a microplate reader (Infinite M200 Pro, Tecan).

### Flow cytometry

Dissociated cells were fixed with a 4% PFA solution in PBS. The cells were blocked and permeabilized with 10% NGS in PBST for 1 h at room temperature. The cells were then incubated with the primary antibody in PBST containing 2% NGS. After washing three times with PBST, the cells were incubated with secondary antibodies for 3 h at room temperature. The samples were analyzed using flow cytometry (FACS Aria III, BD Biosciences).

### Statistical analysis

All results are expressed as the mean ± standard error of the mean (SEM). Differences between the mean values were analyzed using the Student’s *t*-test and were considered statistically significant at *P* < 0.05.

## Results

### Establishment of the AD hPSC line

Since the formation (or differentiation) of organoids is a dynamic process that changes the cellular phenotype over a long period, it is necessary to maintain stable gene expression during this process. Empirically, it is known that the expression of genes introduced into hPSCs weakens or disappears during differentiation. Therefore, we decided to assess the activity of four types of promoters, *CAG, PGK, EF1α*, and *CMV*, which are widely used for in vitro and in vivo gene overexpression, to select an appropriate promoter to drive persistent gene expression during CO formation. To evaluate the activity of various promoters, we constructed four types of *plasmids*, *CAG-mCherry* (CmC), *EF1α-mCherry* (EmC), *PGK-mCherry* (PmC), and *CMV-mCherry* (CMmC), and generated lentiviral particles, as described in the Methods section. *We then established four hESC lines by transfection with the four generated lentiviral particles, each carrying a different promoter*, followed by selection based on the mCherry expression (Fig. S1a and S1b). The COs were generated using these cell lines, and the mCherry expression was serially monitored during CO formation. As shown in Fig. S1c, mCherry expression was commonly observed in the CmC, EmC, PmC, and CMmC COs from day 0 to day 10; however, this expression disappeared on day 20 in the EmC, PmC, and CMmC COs. Strong mCherry expression was stably maintained until day 180 of differentiation in the CmC COs. Although the mechanisms involved in this phenomenon have not been determined, we found that only the *CAG* promoter maintained the stable gene expression from the hPSC stage during the process of CO formation. Through this simple finding, we established a condition for the stable expression of introduced genes for the entire formation process of hPSC-derived COs. We believe that this is a simple but highly valuable finding that will prevent unnecessary trial-and-error experiments in future hPSC-derived organoid studies. The CAG promoter was selected for further experiments.

**Fig. 1:**
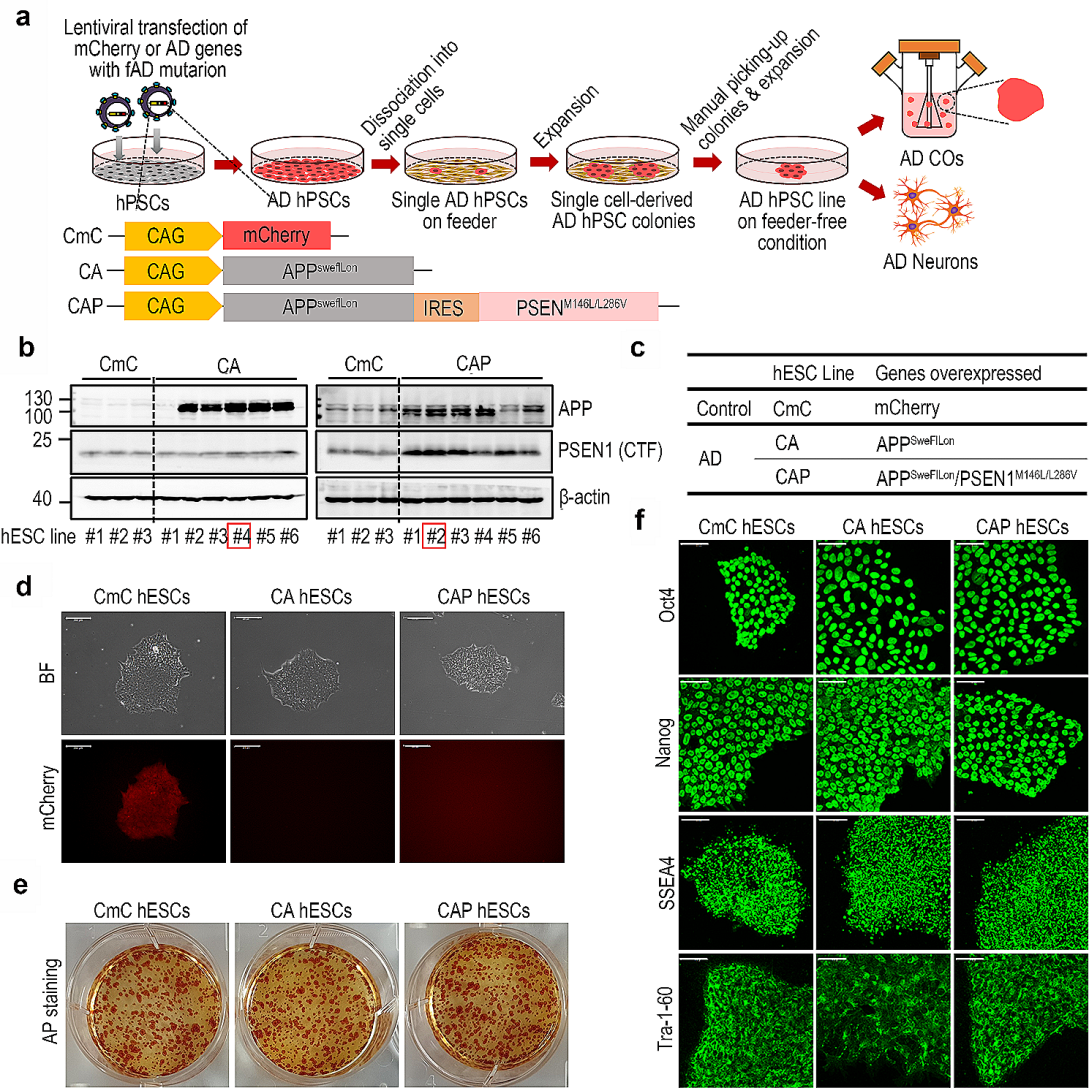
Establishment of human embryonic stem cell (hESC) lines with mCherry or familial Alzheimer’s disease (fAD) mutations. (**a**) Schematic diagram showing the procedures for the establishment of the Alzheimer’s disease (AD) human pluripotent stem cell (hPSC) lines and the generation of AD hPSC-derived AD cerebral organoids (COs) and AD neurons. hPSCs were stably transfected with lentiviral vectors containing mCherry or AD genes with fAD mutations. Dissociated single cells were obtained and plated onto the feeder layer. After expansion, single cell-derived colonies were manually selected, and each hPSC line was established by separate expansion. AD COs or AD neurons were generated from each hPSC line. CAG; CMV early enhancer/chicken β-actin. (**b**) The levels of amyloid precursor protein (APP) and Presenilin1 (PSEN1) expression in the CmC, CA, or CAP hESC lines were determined by western blot analysis. Red squares represent the selected CA or CAP hESC lines based on their APP and/or PSEN1 expression levels. (**c**) Table summarizing the control and AD hESC lines established in this study. (**d**) Representative fluorescence microscope images of the CmC, CA, and CAP hESCs. Scale bar = 200 μm. (**e**) Images showing the alkaline phosphatase activity in the CmC, CA, and CAP hESCs. (f) Representative immunofluorescence images representing the expression of Oct4, Nanog, SSEA4, and Tra-1-60 in the CmC, CA, and CAP hESCs. Scale bar = 100 μm

The fAD genes, human amyloid precursor protein (*APP*) with K670/M671L (Swedish), I176V (Florida), and V717I (London) (APP^SweFlLon^), and *PSEN1* with M146L/L286V mutations (PSEN1^M146L/L286V^) were cloned from the brain tissue of 8-month-old 5XFAD mice, as described in the Methods section. First, lentiviral constructs were generated and designed to express APP^SweFlLon^-only or APP^SweFlLon^ and PSEN1^M146L/L286V^, as previously reported [7, 18], under the control of the CAG promoter. After the generation of CAG-APP^SweFlLon^ (CA) and CAG-APP^SweFlLon^-IRES-PSEN1^M146L/L286V^ (CAP) lentiviral particles, hESCs were transfected with the CA or CAP lentiviral particles. Six-single cell-derived CA or CAP hESC colonies were manually picked and expanded, as shown in Fig. 1a. Among the six hESC lines, one CA hESC line (#4) was selected based on the APP expression level and one CAP hESC line (#2) based on the APP and PSEN1 expression levels by western blotting (Fig. 1b and d). AP staining and immunofluorescence staining of hESC markers, including Oct4, Nanog, SSEA4, and Tra-1-60, showed that the undifferentiation of each chosen CA and CAP hESC line was not affected by the fAD gene transduction (Fig. 1e and f). These results demonstrate the successful establishment of the two AD hESC lines, CA and CAP. Subsequent studies were conducted using these two lines with CmC hESCs as the controls. *To investigate the integration sites and number of copies of the integrated vector, we analyzed the CAP hESCs by whole-genome sequencing analysis. As shown in Fig.**S2**a and**S2**b, we confirmed a single copy insertion in chromosome 17.*

### Robust expression of AD pathologies in the AD COs

**Fig. 2:**
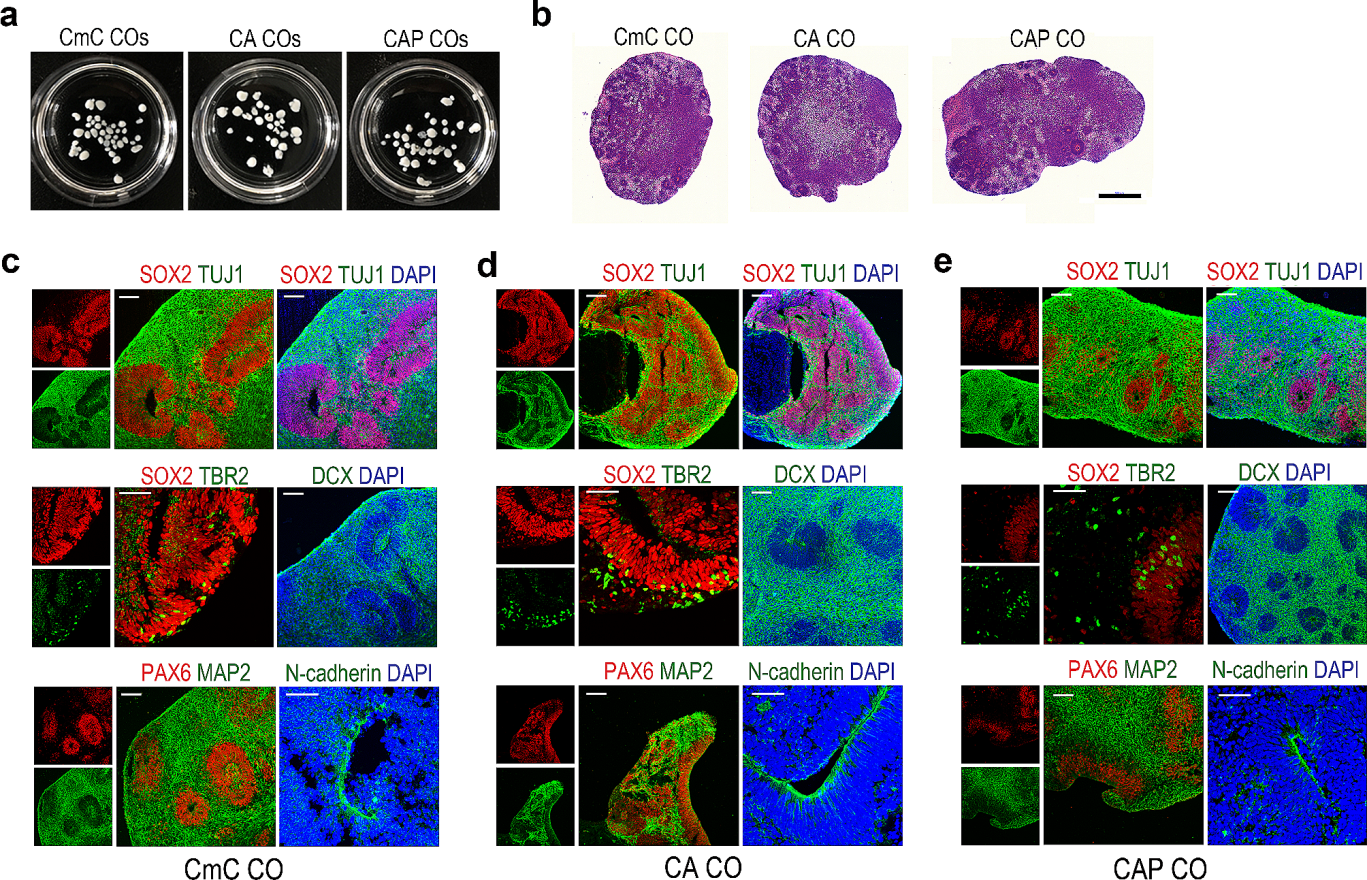
Characterization of cerebral organoids (COs) generated from CmC, CA, and CAP human embryonic stem cell (hESC) lines. (**a**) Seventy-day-old CmC, CA, and CAP COs derived from the CmC, CA, and CAP hESC lines, respectively. (**b**) Representative hematoxylin and eosin-stained sections from the CmC, CA, and CAP COs. Scale bar = 500 μm. (**c-e**) Immunohistochemistry staining for various neuronal markers in the CmC, CA, and CAP COs: SOX2 (red), TUJ1 (green), TBR2 (green), PAX6 (red), MAP2 (green), DCX (green), and neural-specific N-cadherin (green). DAPI indicates the nuclei location (blue). Scale bar = 100 μm

To confirm whether normal CO formation was achieved in our AD hESC lines, we examined the expression of the regional brain markers using immunohistochemical staining with 70-day-old AD (CA and CAP) and control (CmC) COs (Fig. 2a and b). As shown in Fig. 2c and e, histological analysis revealed that various brain region markers, including sex-determining region Y-box 2 (SOX2, a neural progenitor marker), neuron-specific class III beta-tubulin 1 (Tuj1, a neuronal marker), T-box brain protein 2 (TBR2, an intermediate progenitor marker), doublecortin (DCX, a neuronal marker), paired box 6 (PAX6, a radial glial stem cell marker), microtubule-associated protein 2 (MAP2, a neuronal marker), and neural-specific N-cadherin, were commonly expressed in CA and CAP COs, as well as in the CmC control COs, as previously reported [19]. Based on these results, we concluded that normal CO formation was not affected by the transduction of fAD genes, and the subsequent experiments were performed. *To analyze the cell types that the COs were composed of, scRNA-seq analysis was performed. scRNA-seq of enzymatically digested 70-day-old CAP CO revealed the presence of a heterogenous mixture of cells and cell types were divided into 17 clusters, including radial glial cells, neuroepithelial cells, neuronal progenitor cells, intermediate progenitor cells, astrocytes, microglial cells, endothelial cells, pericytes, and five types of distinct neurons (Fig.**S3**).*

**Fig. 3:**
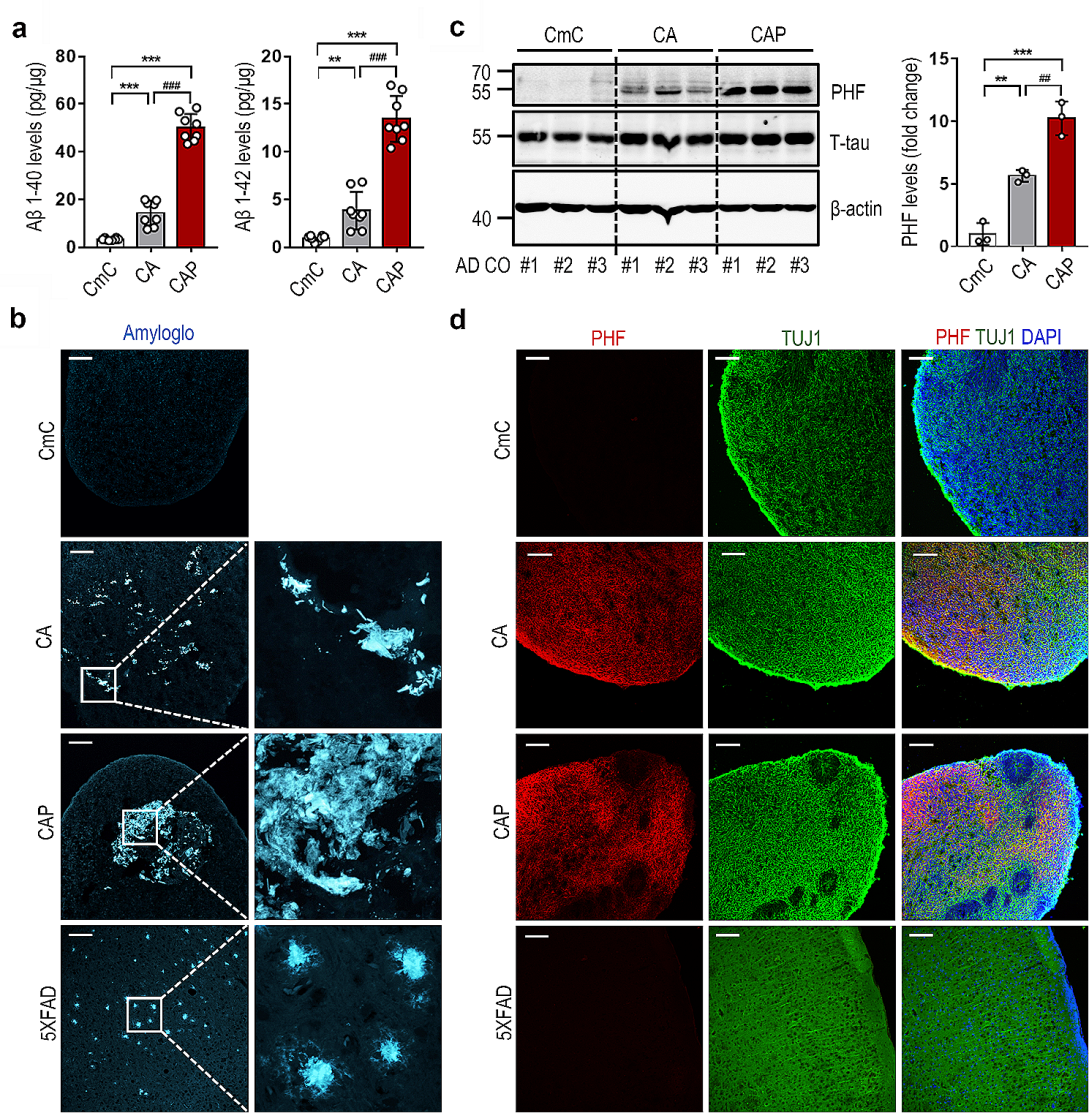
Robust increases of amyloid-β (Aβ) and paired helical filament (PHF)-tau levels in the Alzheimer’s disease (AD) cerebral organoid (CO) model. COs were subjected to analysis by ELISA, western blotting, Amylo-Glo staining, and immunostaining at day 70. (**a**) Aβ1–40 and Aβ1–42 levels in the lysates of CmC, CA, and CAP COs. The data represents the mean ± standard error of the mean (SEM); **P < 0.005 and ***P < 0.001 vs. CmC; ^###^*P* < 0.001 vs. CA; *n* = 8 per sample. (**b**) Detection of amyloid plaques in the CA and CAP COs with Amylo-Glo staining. Scale bar = 100 μm. (**c**) Western blot analysis of the PHF-tau and total tau levels in the CmC, CA, and CAP COs in the lysates of each CO. The data represents the mean ± SEM; **P < 0.005 and ***P < 0.001 vs. CmC; ^##^*P* < 0.005 vs. CA; *n* = 3 per sample. (**E**) Representative images of the immunohistochemistry showing the PHF-tau and TUJ1 in the CmC, CA and, CAP COs. Scale bar = 100 μm

**Fig. 4:**
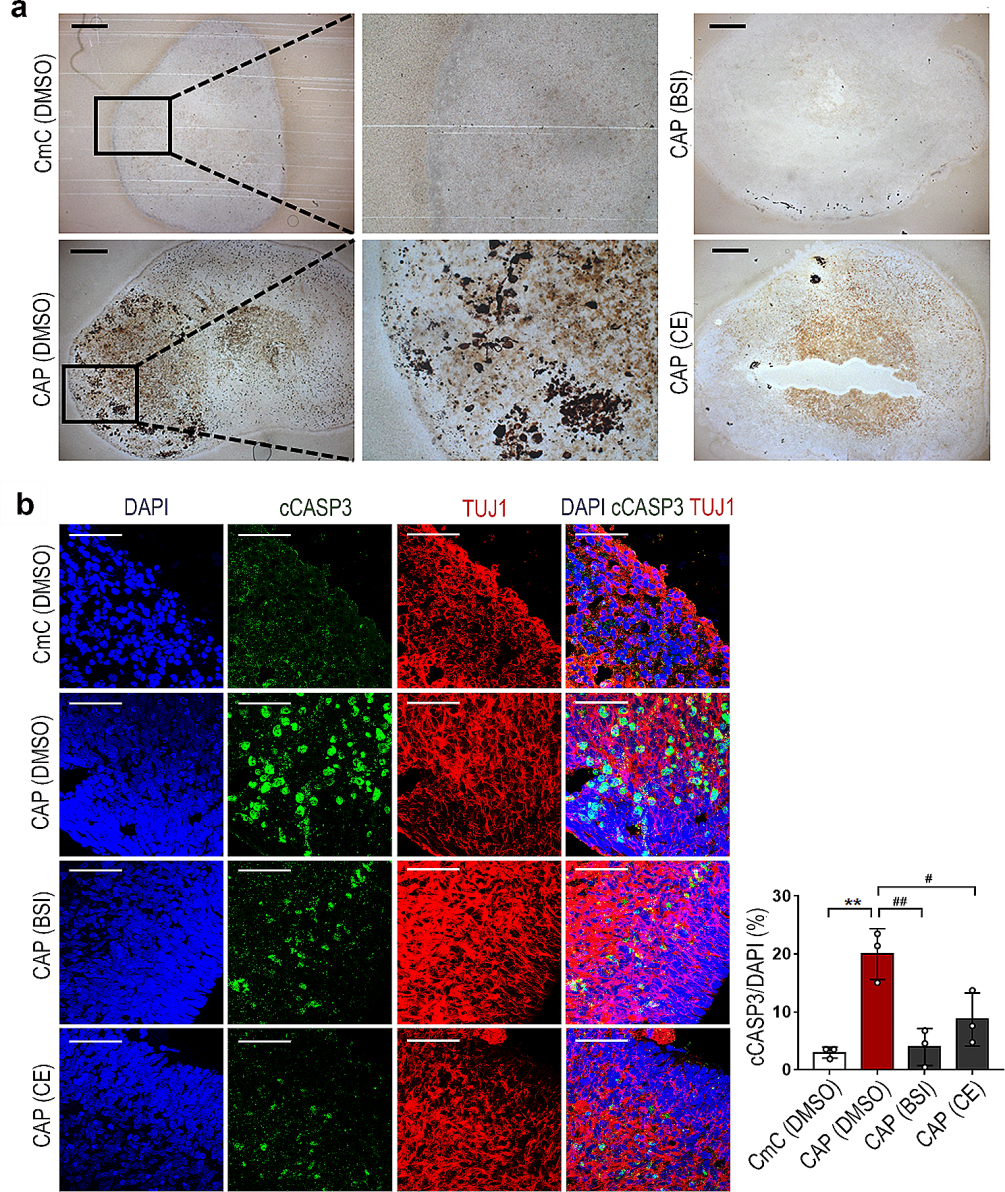
Formation of neurofibrillary tangle (NFT)-like structures and cellular apoptosis in an Alzheimer’s disease (AD) cerebral organoid (CO) model. (**a**) Representative images showing the NFT-like structures detected using Bielschowsky’s silver staining in the CmC and CAP COs treated with or without β-secretase inhibitor (BSI) and compound E (CE). Scale bar = 100 μm. (**b**) Representative immunohistochemistry images showing the cleaved caspase-3 (cCASP3) in the CmC and CAP COs treated with or without BSI or CE

Next, the expression of the AD pathological features, Aβ, and Tau phosphorylation in the AD CO models was analyzed. First, the levels of Aβ 1–40 and 1–42 isoforms in the 70-day-old control and AD COs were measured. As shown in Fig. 3a, the levels of Aβ 1–40 and 1–42 were found to be significantly increased in the CA and CAP COs compared with the CmC control COs. Additionally, the CAP COs showed significantly higher levels of Aβ 1–40 and 1–42 compared to the CA COs. These results were confirmed by histochemical analyses. To detect Aβ deposits, the CA and CAP COs were stained with Amylo-Glo, a fluorescent amyloid-specific dye. As shown in Fig. 3b, robust Aβ accumulation was observed in the CA and CAP COs. Staining with another amyloid-specific dye, thioflavin-S, and immunohistochemistry with 6E10 and 4G8 anti-Aβ antibodies confirmed a robust increase in Aβ accumulation in the CAP COs (Fig. S4a–S4c). Hyperphosphorylation of Tau protein and the formation of NFTs are other pathological markers of AD [20]. As shown in Fig. 3c, western blot analysis revealed a marked increase in PHF-tau in the CA and CAP COs. PHFs are structural constituents of NFTs in AD and are comprised of hyperphosphorylated forms of the microtubule-associated PHF-tau [21]. Similar to Aβ, the PHF-tau levels were found to be significantly increased in the CA and CAP COs compared with the CmC control COs, and the CAP COs showed significantly higher levels than the CA COs. Despite the difference in the PHF-tau expression levels between CA and CAP COs, immunohistochemical analysis showed that PHF-tau was expressed throughout the entire COs in both the CA and CAP COs (Fig. 3d). In addition, Bielschowsky’s silver staining showed strong NFT-like silver deposition in the CAP COs but not in the control CmC COs (Fig. 4a). 5XFAD mice rarely recapitulate tau pathology despite their aggressive AD phenotypes, including Aβ accumulation [22]. Indeed, our results showed that PHF-tau expression was not observed in the brain tissue of 5XFAD mice [23], despite the presence of extensive Aβ plaques (Fig. 3b and d). This result is a typical example of interspecies differences and disproves the importance of developing a human AD model. Moreover, we found that PHF-tau was almost not expressed in the undifferentiated CmC, CA, and CAP hESC lines, with no significant difference between the hESC lines, whereas it had a significantly higher expression in CAP COs (Fig. S5a). In addition, PHF-tau expression was not observed in the PAX6-positive neural progenitor region inside the CAP COs (Fig. S5b). These results indicate that tau phosphorylation is expressed in fully differentiated neuronal cells or tissues in AD COs. Because the expression of genes involved in AD pathology was higher in the CAP COs than in the CA COs, subsequent experiments were conducted using CAP hESCs.

*Meanwhile, we compared the AD phenotypes between the 70-day-old COs generated from wild-type hESCs and early-onset AD patient-derived hiPSCs. In the ELISA experiments, we observed no significant differences in the Aβ1–40 and Aβ1–42 levels between the two groups (Fig.* *S6* *a). In addition, immunohistochemistry revealed no significant differences in PHF-tau expression between the two groups (Fig.* *S6* *b). Based on these results, we provisionally concluded that even if COs are generated from AD patient-derived hiPSCs, it may not be sufficient to demonstrate AD pathologies and will behave similarly to the wild-type cells in the absence of further considerations. This may be achieved by an extended culture period (at least > 70 days) or accelerated by overexpression, which was the method used in this study. In experiments using hiPSCs, our results showed that the levels of Aβ 1–40 and 1–42 were significantly increased in the CAP hiPSC COs compared with the wild-type control hiPSC COs (Fig.* *S7* *a). Immunohistochemistry with 4G8 and 6E10 anti-Aβ antibodies also confirmed the robust increase in Aβ accumulation in CAP hiPSC COs (Fig.* *S7* *b). Western blot analysis further revealed a marked increase in PHF-tau in the CAP hiPSC COs (Fig.* *S7* *c). Consequently, the results of this experiment were the same as those of the hESC experiments. Our AD modeling strategy was to generate COs using hPSCs with the overexpression of fAD genes. In this experiment, we confirmed that our strategy could be applied equally, regardless of the cell type.*

### Application of AD COs in the evaluation of drugs

**Fig. 5:**
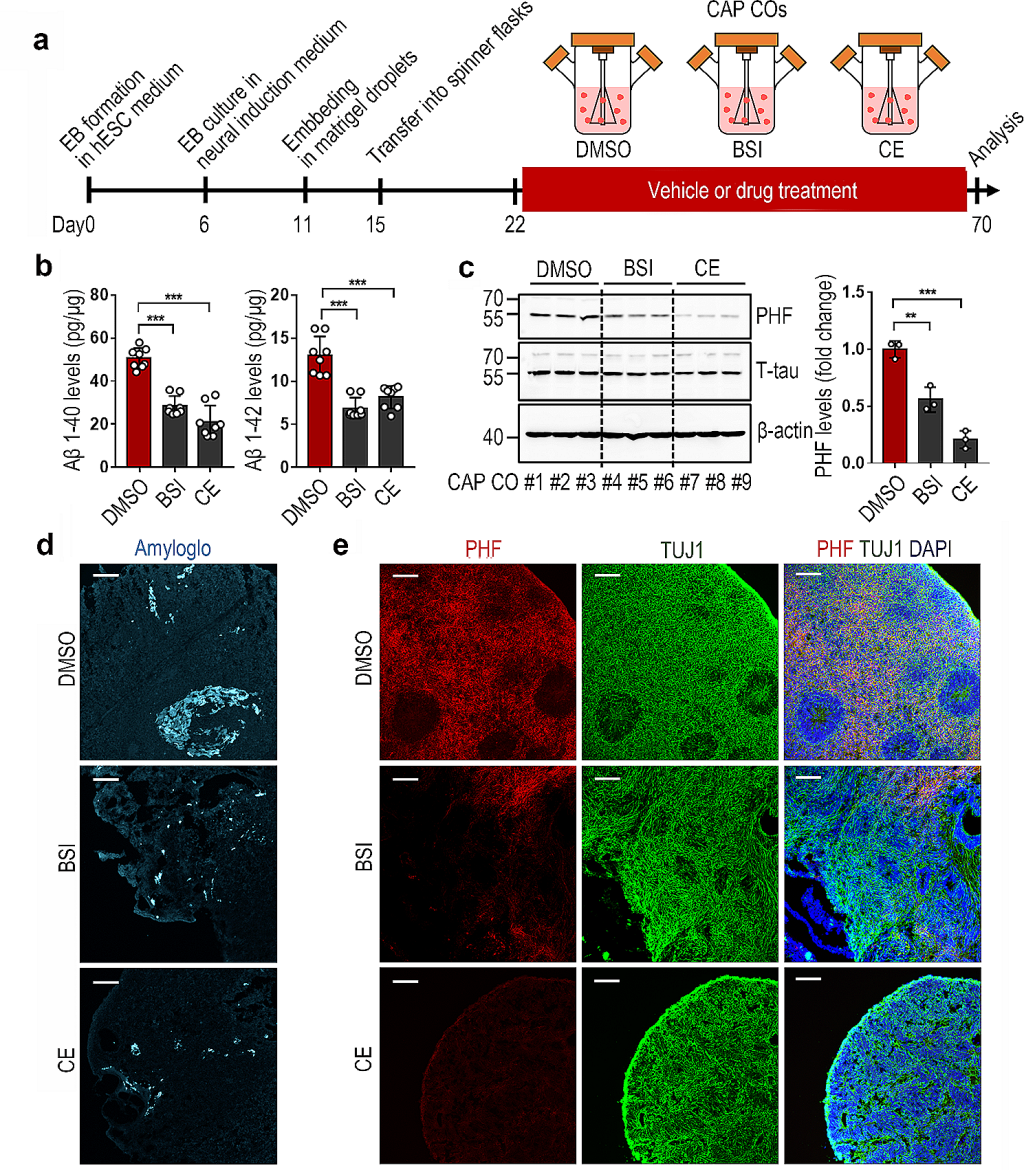
Use of the Alzheimer’s disease (AD) cerebral organoid (CO) model for the evaluation of drug activity. COs were subjected to analysis by ELISA, western blotting, Amylo-Glo staining, and immunostaining at day 70. (**a**) Schematic diagram of the process of CO generation and drug treatment. (**b**) Effect of β-secretase inhibitor (BSI) and compound E (CE) treatment on the levels of Aβ1–40 and Aβ1–42 in the CAP COs. The data represents the mean ± standard error of the mean (SEM); ***P < 0.001 vs. DMSO; *n* = 8 per sample. (**c**) Detection of amyloid plaques in the CAP COs treated with or without BSI or CE using Amylo-Glo staining. Scale bar = 100 μm. (**d**) Effect of BSI and CE treatment on the levels of paired helical filament (PHF) tau and total tau in the CAP COs analyzed by western blot analysis in the lysates of each CO. The data represents the mean ± SEM; **P < 0.005 and ***P < 0.001 vs. DMSO; *n* = 3 per sample. (**e**) Representative immunohistochemistry images showing the PHF-tau and TUJ1 in the CAP COs treated with or without BSI or CE. Scale bar = 100 μm

To determine the usefulness of the AD CO model as a tool for drug testing, CAP COs were treated with BACE1 inhibitor IV (BSI), a β-secretase inhibitor, or compound E (CE), a γ-secretase inhibitor (Fig. 5a). Two aspartic proteases, β-secretase and γ-secretase, mediate Aβ production via APP cleavage at different sites [24]. Our results showed that treatment with BSI or CE markedly decreased the levels of Aβ 1–40 and 1–42 in the CAP COs (Fig. 5b). Western blot analysis revealed that the high expression of PHF-tau in the CAP COs was also attenuated by BSI or CE treatment (Fig. 5c). Immunohistochemistry confirmed the decreased Aβ and PHF-tau expression following BSI or CE treatment in the CAP COs (Fig. 5d and e). In addition, the increase in NFT-like structures in CAP COs was attenuated by BSI or CE treatment (Fig. 4a). These results suggest that the well-known theoretical background in which tau phosphorylation is driven by the excessive expression of Aβ is reflected in our AD CO model.

To investigate whether the neurodegenerative features of AD were simulated in CAP COs, cleaved caspase-3 (cCASP3), a well-known apoptosis marker, was analyzed by immunohistochemistry. Increased cCASP3 immunoreactivity was observed in the CAP CO compared with that in the CmC CO, which was significantly reduced in the CAP COs by BSI or CE treatment (Fig. 4b). cCASP3-positive cells were counted on the surface of each CO to exclude the possible influence of a necrotic region in the core of the CO. These results suggest that the cellular apoptosis observed in the CAP CO is closely related to the excessive formation of Aβ. Taken together, our AD CO model is a useful tool for drug development studies.

**Fig. 6:**
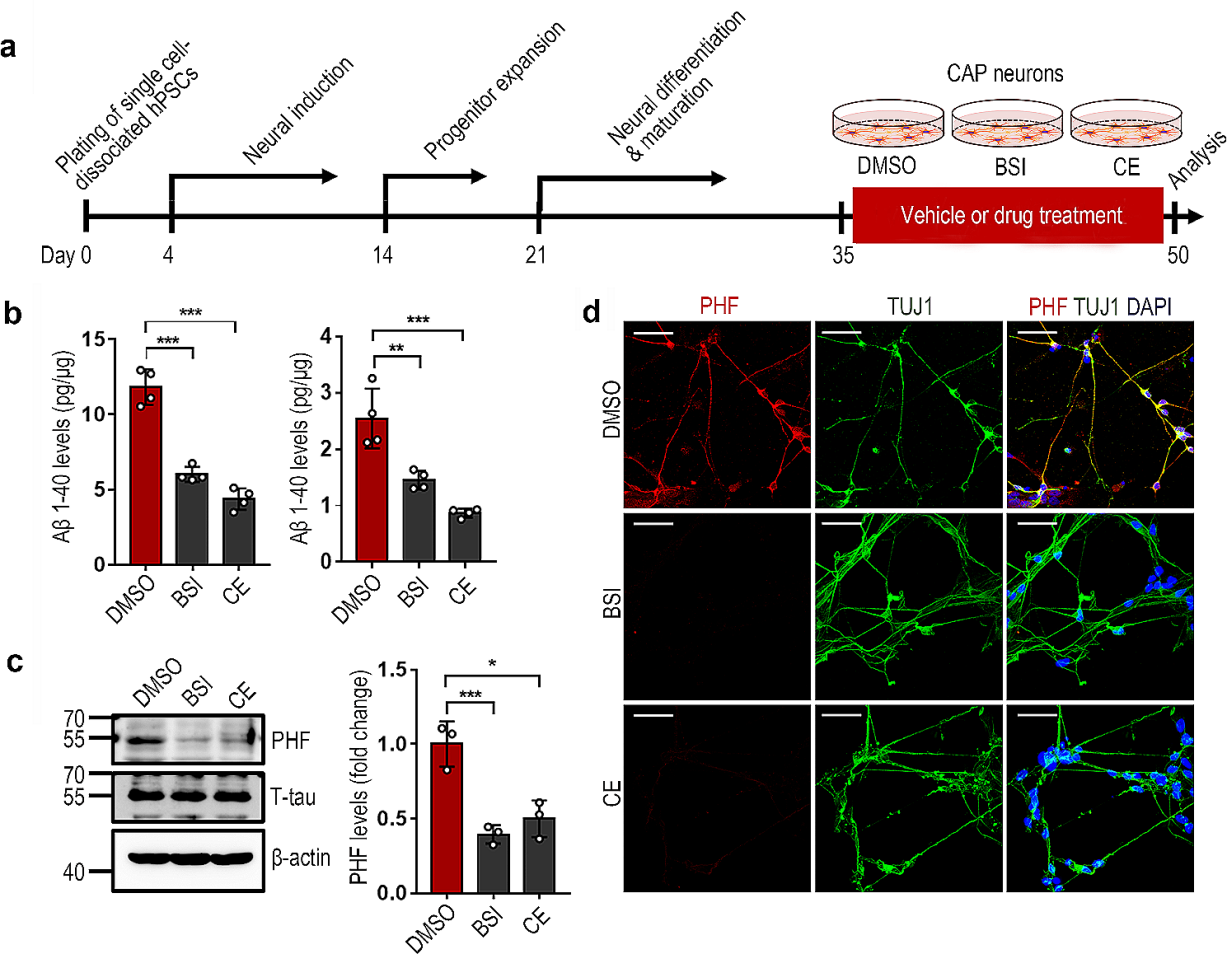
Use of Alzheimer’s disease (AD) neurons for the evaluation of drug activity. AD neurons were subjected to analysis by ELISA, western blotting, and immunostaining at day 50. (**a**) Schematic representation of the process of neuronal differentiation and drug treatment. (**b**) Effect of β-secretase inhibitor (BSI) and compound E (CE) on the levels of Aβ1–40 and Aβ1–42 in the lysates of CAP neurons. The data represents the mean ± standard error of the mean (SEM); ***P < 0.001 vs. DMSO; *n* = 4 per sample. (**c**) Effect of BSI and CE treatment on the levels of paired helical filament (PHF) tau and total tau in the CAP neurons analyzed by western blot analysis of the lysates in each neuron. The data represents the mean ± SEM; *P < 0.05 and ***P < 0.001 vs. DMSO; *n* = 3 per sample. (**d**) Representative immunostaining images of the PHF-tau and TUJ1 in the CAP neurons treated with or without BSI or CE. The bar graph represents the ratio of cleaved caspase-3 (cCASP3)-positive cells to the total number of cells as measured by DAPI staining. Mean ± SEM; **P < 0.005 vs. CmC (DMSO); ^#^*P* < 0.05 and ^##^*P* < 0.005 vs. CAP (DMSO); *n* = 3 per sample

We further hypothesized that if an AD CO can be generated from AD hPSCs, AD neurons could also be produced by the neuronal differentiation of AD hPSCs, and AD neurons could be used as a 2D AD model. We first induced neuronal differentiation from the CmC control and CAP hESC lines using a modified version of a previously reported protocol [25, 26]. As shown in Fig. S8a, the flow cytometry results revealed that > 70% of the differentiated cells were TUJ1-positive in both the hESC lines under differentiation conditions, and these cells strongly expressed neuronal markers, including MAP2 and DCX, as well as TUJ1 (Fig. S8b and S8c). ELISA analysis demonstrated that the Aβ 1–40 and 1–42 levels were significantly increased in the CAP neuronal cells compared with those in the CmC control (Fig. S9a). Western blot analysis revealed that the PHF-tau expression levels were also markedly high in the CAP neuronal cells (Fig. S9b). Although Aβ plaque expression was not detected under these culture conditions, a strong PHF-tau expression in the TUJ1-positive CAP neuronal cells was confirmed using immunocytochemistry (Fig. S9c). To determine the usefulness of the AD neuronal cell model as a tool for drug testing, CAP neuronal cells were treated with BSI or CE (Fig. 6a). In this experiment, the Aβ 1–40 and 1–42 levels were found to be markedly inhibited by BSI and CE treatment in the CAP neuronal cells (Fig. 6b). Western blotting and immunocytochemistry showed that the PHF-tau levels were also significantly attenuated by BSI or CE treatment in these cells (Fig. 6c and d). These results suggest that our transgenic AD hPSCs can be used for 3D AD modeling through CO generation or 2D AD modeling through differentiation into neuronal cells, and these models can be used as tools for drug testing.

## Discussion

CO models have unlocked the prospect of answering many questions regarding various brain diseases [16, 27–30]. An AD CO model can thus be used to explore the mechanisms underlying AD pathogenesis and to develop drugs for the treatment or prevention of AD [6]. In this study, we report on a transgenic hPSC-derived AD CO modeling technology. We found that an AD CO disease model could be established from normal hPSCs using simple genetic manipulation. We generated clonal AD hPSC lines overexpressing the fAD mutant *APP* gene (*APP*^*SweFlLon*^) (CA) or the fAD mutant *APP* and *PSEN1* genes (*PSEN1*^*M146L/L286V*^) (CAP) using the *CAG* promoter. According to recent reports, AD biomarkers are classified into Aβ deposition, pathological tau expression, and neurodegeneration (the ATN framework) [31]. Therefore, replication of ATN pathology is required to generate an appropriate disease model for AD. In our AD COs, significantly elevated expression and accumulation of Aβ were detected. Our results specifically showed that Aβ was increased by the overexpression of mutant *APP* alone (CA) but was highly promoted by the co-expression with mutant *PSEN1* (CAP). Another pathological hallmark of AD is tau hyperphosphorylation. The hyperphosphorylation of tau protein leads to the generation of PHFs and NFTs, which are key neuropathological features of AD and tauopathies [32, 33]. Consistent with the Aβ results, PHF-tau was increased by the overexpression of mutant *APP* and was highly promoted by the co-expression of mutant *PSEN1*. In addition, the expression of NFT-like structures was strongly enhanced by the overexpression of mutant *APP* and *PSEN1*. In addition, AD CO showed a marked increase in the cellular apoptotic marker, cCASP3. Neurodegeneration is another pathological feature of AD, and although the underlying mechanism remains elusive, analyses of postmortem histology from patients and studies using experimental animal and cell culture models have implicated neuronal apoptosis [34]. Therefore, our results provide clear evidence that our model replicates the ATN biomarkers and indicate that our AD modeling strategy was effective.

Organoid technology has paved the way for the discovery of new therapeutic drugs by providing a platform for drug screening [35]. Organoid models of microcephaly, cystic fibrosis, ulcerative colitis, and Crohn’s disease have already aided in the development of new pharmaceuticals for these diseases [36, 37]. In this study, we evaluated our AD CO model as a useful platform for the development of novel AD treatment drugs. Because increased levels of Aβ cause aggregation, which is responsible for the formation of plaques and the induction of tau pathology, it leads to cell death and neurodegeneration; therefore, Aβ is the primary target for AD [38, 39]. Aβ peptides are formed after the sequential cleavage of the amyloid precursor protein (APP), catalyzed by β-site APP-cleaving enzyme 1 (BACE1, β-secretase) and γ-secretase [40, 41]. Preclinical studies on the development of drugs targeting BACE1 and γ-secretase have been conducted [42, 43]. Our results revealed that the levels of Aβ, PHF-tau, NFT-like structures, and cellular apoptosis were significantly attenuated by the treatment with BACE1 inhibitor IV (BSI), a BACE 1 inhibitor, and compound E (CE), a γ-secretase. These results provide direct evidence for the usefulness of our AD CO model in drug screening studies.

*Recently, several studies have reported CO models established from hiPSCs obtained from patients with AD, and these models showed AD pathology* [2, 44, 45]. *However, we found that even if the COs are generated from hiPSCs derived from patients with AD, it may not be sufficient to demonstrate AD pathology. In addition, there is a need for the cumbersome process of obtaining tissue samples from the patient, followed by establishing a hiPSC line from the sample. In this study, we demonstrated that our AD CO model, derived from a normal hPSC line using a simple overexpression strategy of fAD genes, showed robust AD pathology within a relatively short period of time. Reproducibility was also demonstrated in an experiment using hiPSCs. Further, we demonstrated the usefulness of our AD CO model as a tool for drug screening.*

Despite the disadvantage of not being able to observe histological features, the 2D cellular model remains useful. Various 2D in vitro AD models have produced valuable results that contributed to the understanding of disease mechanisms by replicating aspects of AD pathology at the cellular level [46–49]. Differentiated neuronal subtypes have been used for multiple small-scale screenings of compounds that target disease-related pathways in AD [50–53]. In a recent study, over 1600 compounds were screened for the reduction of tau phosphorylation in neuronal models, which identified numerous hits and ultimately advanced our understanding of the biology underlying phosphorylated tau accumulation [54]. In the present study, neuronal cells differentiated from the AD hPSC line used for the generation of AD COs were found to robustly express Aβ and phosphorylated tau, and the levels of Aβ and phosphorylated tau were significantly attenuated by the treatment with BSI and CE. These results indicate that our transgenic AD hPSC line can also be used as a 2D neuronal AD model. If the scope is further expanded, this model may be used as another type of cellular model using technologies for hPSC differentiation into other cell types.

## Conclusion

COs represent an excellent in vitro model of the genetic forms of AD and may enable the study of AD pathology at more physiological levels. Future experiments using this technology are likely to enable real-time studies such as cellular reactions to the accumulation of protein aggregates, the relationship between amyloid and NFTs, and the proteins and signaling pathways altered by the formation of protein deposits etc., to provide novel therapeutic targets.

Various genetic and environmental factors are involved in the development of AD. Our modeling strategy, in which the pathology of AD in COs and neuronal cells was replicated using transgenic hPSCs, can be used to study the function of various genetic variants, thus enabling the development of new AD models. It is worth mentioning that this strategy is not limited to AD or other types of central nervous system diseases but can also serve to promote the development of various in vitro models for various human diseases and drug screening tools using organoid technology.

### Study limitations

*COs reflect the histological and functional features of the brain, which renders them suitable models for neurological diseases. They contain diverse cell types, including neurons and glial cells, similar to those that are found in the human brain. Nevertheless, COs have obvious limitations. In addition to the replicated AD pathologies in the AD CO model in this study, more diverse pathologies, such as cerebral amyloid angiopathy (CAA) and neuroinflammation, are observed in the brains of patients with AD* [55]. *However, a lack of vasculature and a deficiency in immune components in the current COs restrict their use in modeling CAA or neuroinflammatory responses* [56]. *The immaturity of COs also remains a challenge that needs to be overcome* [57]. *COs represent the fetal stage of brain development and may not fully replicate the characteristics of AD, which typically manifest in adults. In addition, COs are induced through unguided methods to promote spontaneous differentiation, which can result in batch-to-batch variability, thus potentially compromising reproducibility* [17]. *Despite these limitations, COs remain the only viable human model as an alternative to animal disease models. Although various efforts are underway to overcome these limitations, AD CO can be used as an improved model in the future.*

### Electronic supplementary material

Below is the link to the electronic supplementary material.

Supplementary Material 1

Supplementary Material 2

Supplementary Material 3

Supplementary Material 4

Supplementary Material 5

Supplementary Material 6

Supplementary Material 7

Supplementary Material 8

Supplementary Material 9

Supplementary Material 10

Supplementary Material 11

## Data Availability

The data that support the findings of this study are available from the corresponding author upon reasonable request.

## Abbreviations

AD: Alzheimer’s disease
Aβ: Amyloid β
APP: Amyloid precursor protein
bFGF: Basic fibroblast growth factor
CAG: Cytomegalovirus early enhancer/chicken β actin
cDNA: Complementary DNA
CMV: Cytomegalovirus
CO: Cerebral organoid
DCX: Doublecortin
DMEM/F-12: Dulbecco’s Modified Eagle Medium/Nutrient Mixture F-12
EB: Embryoid body
EDTA: Ethylenediaminetetraacetic acid
EF1α: Human elongation factor-1 α
HEK: Human embryonic kidney
hESCs: Human embryonic stem cells
hiPSCs: Human-induced pluripotent stem cells
hPSCs: Human pluripotent stem cells
HRP: Horseradish peroxidase
IgG: Immunoglobulin G
MAP2: Microtubule-associated protein 2
MEM: Minimum essential medium
NFT: Neurofibrillary tangle
NEAA: Nonessential amino acids
OCT4: Octamer-binding transcription factor 4
PAX6: Paired box 6
PBST: 0.1% Triton X-100 in phosphate-buffered saline
PGK: Phosphoglycerate kinase 1
PHF: Paired helical filament
PSEN1: Presenilin1
PVDF: Polyvinylidene fluoride
SDS-PAGE: Sodium dodecyl sulfate–polyacrylamide gel electrophoresis
SEM: Standard error of the mean
shRNA: Short hairpin RNA
SOX2: Sex-determining region Y-box 2
SSEA4: Stage-specific embryonic antigen 4
TBR2: T-box brain protein 2
TUJ1: Neuron-specific class III beta-tubulin 1

## References

[1] Tanzi RE, Bertram L (2005). Twenty years of the Alzheimer’s disease amyloid hypothesis: a genetic perspective. Cell.

[2] Gonzalez C, Armijo E, Bravo-Alegria J, Becerra-Calixto A, Mays CE, Soto C (2018). Modeling amyloid beta and tau pathology in human cerebral organoids. Mol Psychiatry.

[3] Gerakis Y, Hetz C (2019). Brain organoids: a next step for humanized Alzheimer’s disease models?. Mol Psychiatry.

[4] Lee HK, Velazquez Sanchez C, Chen M, Morin PJ, Wells JM, Hanlon EB (2016). Three Dimensional Human Neuro-Spheroid Model of Alzheimer’s Disease Based on Differentiated Induced Pluripotent Stem cells. PLoS ONE.

[5] Ravi M, Paramesh V, Kaviya SR, Anuradha E, Solomon FD (2015). 3D cell culture systems: advantages and applications. J Cell Physiol.

[6] Bi FC, Yang XH, Cheng XY, Deng WB, Guo XL, Yang H (2021). Optimization of cerebral organoids: a more qualified model for Alzheimer’s disease research. Transl Neurodegener.

[7] Choi SH, Kim YH, Hebisch M, Sliwinski C, Lee S, D’Avanzo C (2014). A three-dimensional human neural cell culture model of Alzheimer’s disease. Nature.

[8] Amin ND, Pasca SP. Building Models of Brain Disorders with Three-Dimensional Organoids. Neuron. 2018;100(2):389–405. 10.1016/j.neuron.2018.10.007 10.1016/j.neuron.2018.10.007 30359604

[9] Bagley JA, Reumann D, Bian S, Levi-Strauss J, Knoblich JA (2017). Fused cerebral organoids model interactions between brain regions. Nat Methods.

[10] Luo C, Lancaster MA, Castanon R, Nery JR, Knoblich JA, Ecker JR (2016). Cerebral organoids recapitulate epigenomic signatures of the human fetal brain. Cell Rep.

[11] King A (2018). The search for better animal models of Alzheimer’s disease. Nature.

[12] Sevigny J, Chiao P, Bussière T, Weinreb PH, Williams L, Maier M (2016). The antibody aducanumab reduces Aβ plaques in Alzheimer’s disease. Nature.

[13] Calvin CM, de Boer C, Raymont V, Gallacher J, Koychev I, European Prevention of Alzheimer’s Dementia (EPAD) Consortium (2020). Prediction of Alzheimer’s disease biomarker status defined by the ‘ATN framework’ among cognitively healthy individuals: results from the EPAD longitudinal cohort study. Alzheimers Res Ther.

[14] Li H. Aligning sequence reads, clone sequences and assembly contigs with BWA-MEM. arXiv preprint 2013. 10.48550/arXiv.1303.3997

[15] Hao Y, Stuart T, Kowalski MH, Choudhary S, Hoffman P, Hartman A (2023). Dictionary learning for integrative multimodal and scalable single-cell analysis. Nat Biotechnol.

[16] Becht E, McInnes L, Healy J, Dutertre CA, Kwok IWH, Ng LG et al. Dictionary learning for integrative, multimodal and scalable single-cell analysis. 2019;37:38–44. 10.1038/nbt.4314

[17] Lancaster MA, Knoblich JA (2014). Generation of cerebral organoids from human pluripotent stem cells. Nat Protoc.

[18] Park J, Wetzel I, Marriott I, Dreau D, D’Avanzo C, Kim DY (2018). A 3D human triculture system modeling neurodegeneration and neuroinflammation in Alzheimer’s disease. Nat Neurosci.

[19] Lancaster MA, Renner M, Martin CA, Wenzel D, Bicknell LS, Hurles ME (2013). Cerebral organoids model human brain development and microcephaly. Nature.

[20] Trojanowski JQ, Lee VM (2002). The role of tau in Alzheimer’s disease. Med Clin North Am.

[21] Hanger DP, Betts JC, Loviny TL, Blackstock WP, Anderton BH (1998). New phosphorylation sites identified in hyperphosphorylated tau (paired helical filament-tau) from Alzheimer’s disease brain using nanoelectrospray mass spectrometry. J Neurochem.

[22] Sasaguri H, Nilsson P, Hashimoto S, Nagata K, Saito T, De Strooper B (2017). APP mouse models for Alzheimer’s disease preclinical studies. EMBO J.

[23] Oakley H, Cole SL, Logan S, Maus E, Shao P, Craft J (2006). Intraneuronal beta-amyloid aggregates, neurodegeneration, and neuron loss in transgenic mice with five familial Alzheimer’s disease mutations: potential factors in amyloid plaque formation. J Neurosci.

[24] Ghosh AK, Brindisi M, Tang J (2012). Developing beta-secretase inhibitors for treatment of Alzheimer’s disease. J Neurochem.

[25] Maroof AM, Keros S, Tyson JA, Ying SW, Ganat YM, Merkle FT (2013). Directed differentiation and functional maturation of cortical interneurons from human embryonic stem cells. Cell Stem Cell.

[26] Xiang Y, Kim KY, Gelernter J, Park IH, Zhang H (2015). Ethanol upregulates NMDA receptor subunit gene expression in human embryonic stem cell-derived cortical neurons. PLoS ONE.

[27] Bershteyn M, Nowakowski TJ, Pollen AA, Di Lullo E, Nene A, Wynshaw-Boris A (2017). Human iPSC-Derived cerebral organoids Model Cellular features of Lissencephaly and reveal prolonged mitosis of outer Radial Glia. Cell Stem Cell.

[28] Mariani J, Coppola G, Zhang P, Abyzov A, Provini L, Tomasini L (2015). FOXG1-Dependent dysregulation of GABA/Glutamate neuron differentiation in Autism Spectrum disorders. Cell.

[29] Mellios N, Feldman DA, Sheridan SD, Ip JPK, Kwok S, Amoah SK (2018). MeCP2-regulated miRNAs control early human neurogenesis through differential effects on ERK and AKT signaling. Mol Psychiatry.

[30] Raja WK, Mungenast AE, Lin YT, Ko T, Abdurrob F, Seo J (2016). Self-Organizing 3D human neural tissue derived from Induced Pluripotent stem cells recapitulate Alzheimer’s Disease Phenotypes. PLoS ONE.

[31] Jack CR, Bennett DA, Blennow K, Carrillo MC, Dunn B, Haeberlein SB (2018). NIA-AA Research Framework: toward a biological definition of Alzheimer’s disease. Alzheimers Dement.

[32] Cripps D, Thomas SN, Jeng Y, Yang F, Davies P, Yang AJ (2006). Alzheimer disease-specific conformation of hyperphosphorylated paired helical filament-tau is polyubiquitinated through Lys-48, Lys-11, and Lys-6 ubiquitin conjugation. J Biol Chem.

[33] Santa-Maria I, Varghese M, Ksiezak-Reding H, Dzhun A, Wang J, Pasinetti GM (2012). Paired helical filaments from Alzheimer disease brain induce intracellular accumulation of tau protein in aggresomes. J Biol Chem.

[34] Mattson MP (2000). Apoptosis in neurodegenerative disorders. Nat Rev Mol Cell Biol.

[35] Heydari Z, Moeinvaziri F, Agarwal T, Pooyan P, Shpichka A, Maiti TK (2021). Organoids: a novel modality in disease modeling. Biodes Manuf.

[36] Dutta D, Heo I, Clevers H (2017). Disease modeling in stem cell-derived 3D Organoid systems. Trends Mol Med.

[37] Lancaster MA, Huch M (2019). Disease modelling in human organoids. Dis Model Mech.

[38] Graham WV, Bonito-Oliva A, Sakmar TP (2017). Update on Alzheimer’s Disease Therapy and Prevention Strategies. Annu Rev Med.

[39] van Bokhoven P, de Wilde A, Vermunt L, Leferink PS, Heetveld S, Cummings J (2021). The Alzheimer’s disease drug development landscape. Alzheimers Res Ther.

[40] O’Brien RJ, Wong PC (2011). Amyloid precursor protein processing and Alzheimer’s disease. Annu Rev Neurosci.

[41] Selkoe DJ (2001). Alzheimer’s disease: genes, proteins, and therapy. Physiol Rev.

[42] Moussa-Pacha NM, Abdin SM, Omar HA, Alniss H, Al-Tel TH (2020). BACE1 inhibitors: current status and future directions in treating Alzheimer’s disease. Med Res Rev.

[43] Hur JY (2022). γ-Secretase in Alzheimer’s disease. Exp Mol Med.

[44] Park JC, Jang SY, Lee D, Lee J, Kang U, Chang H (2021). A logical network-based drug-screening platform for Alzheimer’s disease representing pathological features of human brain organoids. Nat Commun.

[45] Vanova T, Sedmik J, Raska J, Cerna KA, Taus P, Pospisilova V (2023). Cerebral organoids derived from patients with Alzheimer’s disease with PSEN1/2 mutations have defective tissue patterning and altered development. Cell Rep.

[46] Armijo E, Gonzalez C, Shahnawaz M, Flores A, Davis B, Soto C (2017). Increased susceptibility to Abeta toxicity in neuronal cultures derived from familial Alzheimer’s disease (PSEN1-A246E) induced pluripotent stem cells. Neurosci Lett.

[47] Israel MA, Yuan SH, Bardy C, Reyna SM, Mu Y, Herrera C (2012). Probing sporadic and familial Alzheimer’s disease using induced pluripotent stem cells. Nature.

[48] Kondo T, Asai M, Tsukita K, Kutoku Y, Ohsawa Y, Sunada Y (2013). Modeling Alzheimer’s disease with iPSCs reveals stress phenotypes associated with intracellular abeta and differential drug responsiveness. Cell Stem Cell.

[49] Mahairaki V, Ryu J, Peters A, Chang Q, Li T, Park TS (2014). Induced pluripotent stem cells from familial Alzheimer’s disease patients differentiate into mature neurons with amyloidogenic properties. Stem Cells Dev.

[50] Wang C, Najm R, Xu Q, Jeong DE, Walker D, Balestra ME (2018). Gain of toxic apolipoprotein E4 effects in human iPSC-derived neurons is ameliorated by a small-molecule structure corrector. Nat Med.

[51] Young JE, Fong LK, Frankowski H, Petsko GA, Small SA, Goldstein LSB (2018). Stabilizing the Retromer Complex in a human stem cell model of Alzheimer’s Disease reduces TAU phosphorylation independently of amyloid precursor protein. Stem Cell Rep.

[52] Kimura J, Shimizu K, Kajima K, Yokosuka A, Mimaki Y, Oku N (2018). Nobiletin reduces intracellular and extracellular beta-amyloid in iPS Cell-Derived Alzheimer’s disease model neurons. Biol Pharm Bull.

[53] Kondo T, Imamura K, Funayama M, Tsukita K, Miyake M, Ohta A (2017). iPSC-Based compound screening and in Vitro trials identify a synergistic anti-amyloid beta combination for Alzheimer’s Disease. Cell Rep.

[54] van der Kant R, Langness VF, Herrera CM, Williams DA, Fong LK, Leestemaker Y (2019). Cholesterol metabolism is a Druggable Axis that independently regulates tau and amyloid-beta in iPSC-Derived Alzheimer’s disease neurons. Cell Stem Cell.

[55] DeTure MA, Dickson DW (2019). The neuropathological diagnosis of Alzheimer’s disease. Mol Neurodegener.

[56] Cakir B, Park IH (2022). Getting the right cells. Elife.

[57] Perez-Nievas BG (2023). Brain organoids fill the gap. Nat Neurosci.

