# Supplementary figures and images for: Simple modeling of familial Alzheimer’s disease using human pluripotent stem cell-derived cerebral organoid technology
Source: Stem Cell Res Ther. 2024 Apr 24;15:118. doi: 10.1186/s13287-024-03732-1 (PMC11040922; doi:10.1186/s13287-024-03732-1)

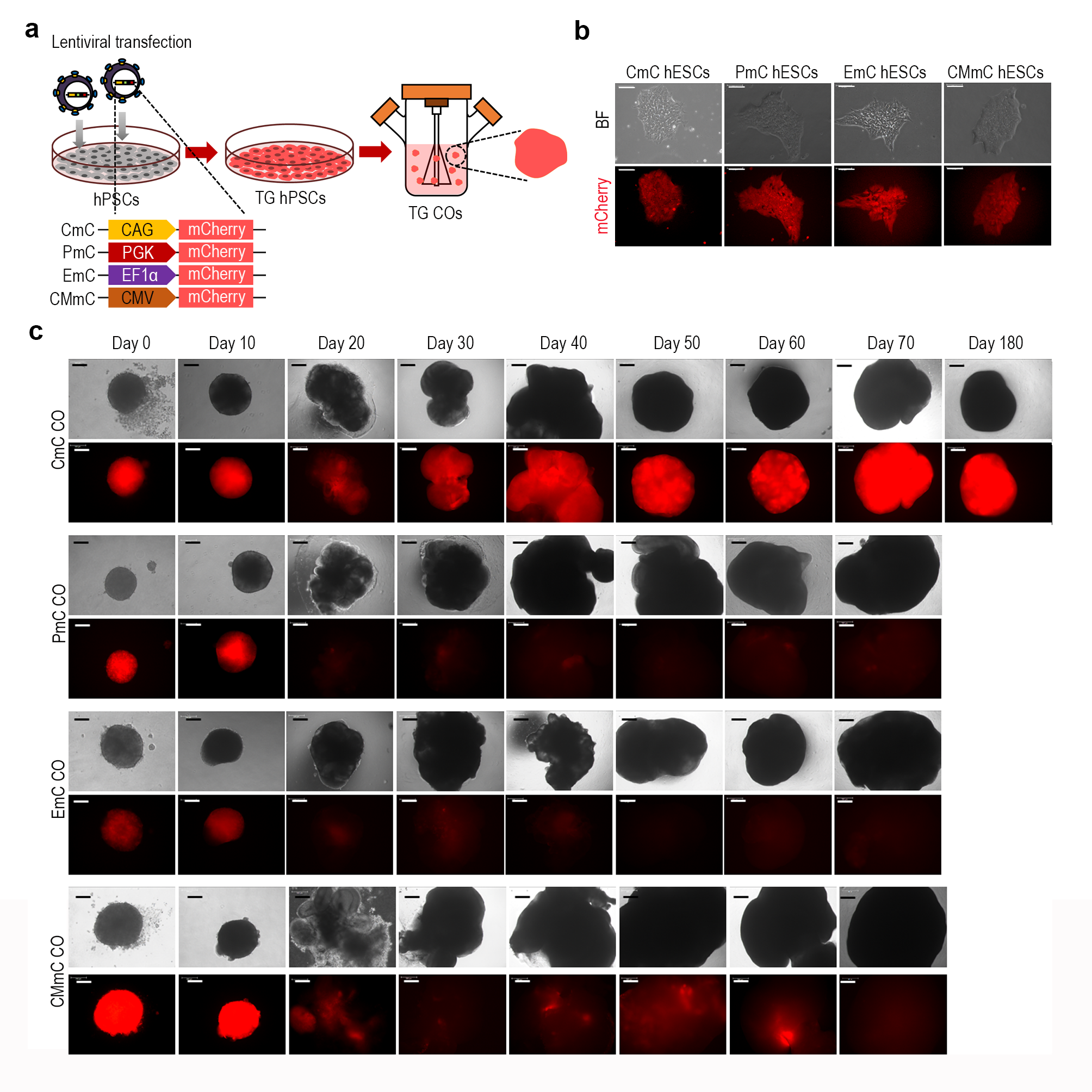

Supplement: Supplementary file 1 — Supplementary Material 1 [file 13287_2024_3732_MOESM1_ESM.png]

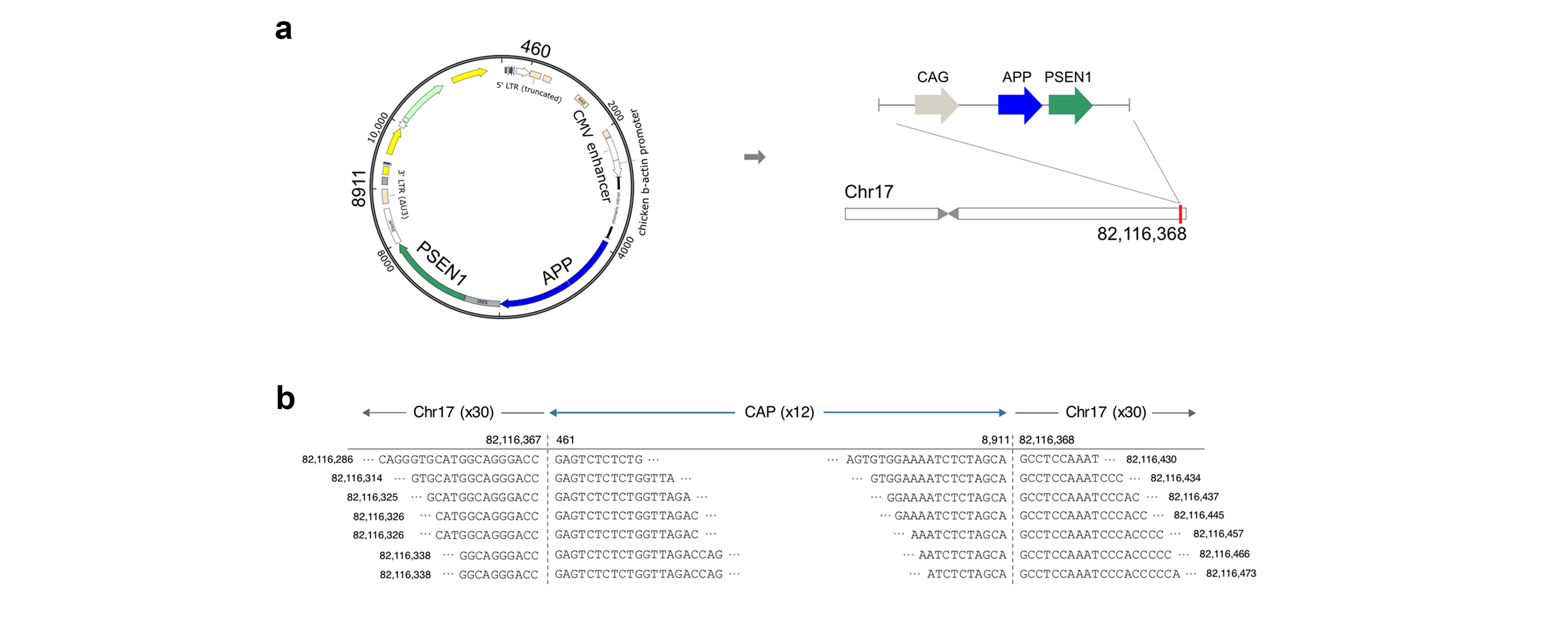

Supplement: Supplementary file 2 — Supplementary Material 2 [file 13287_2024_3732_MOESM2_ESM.png]

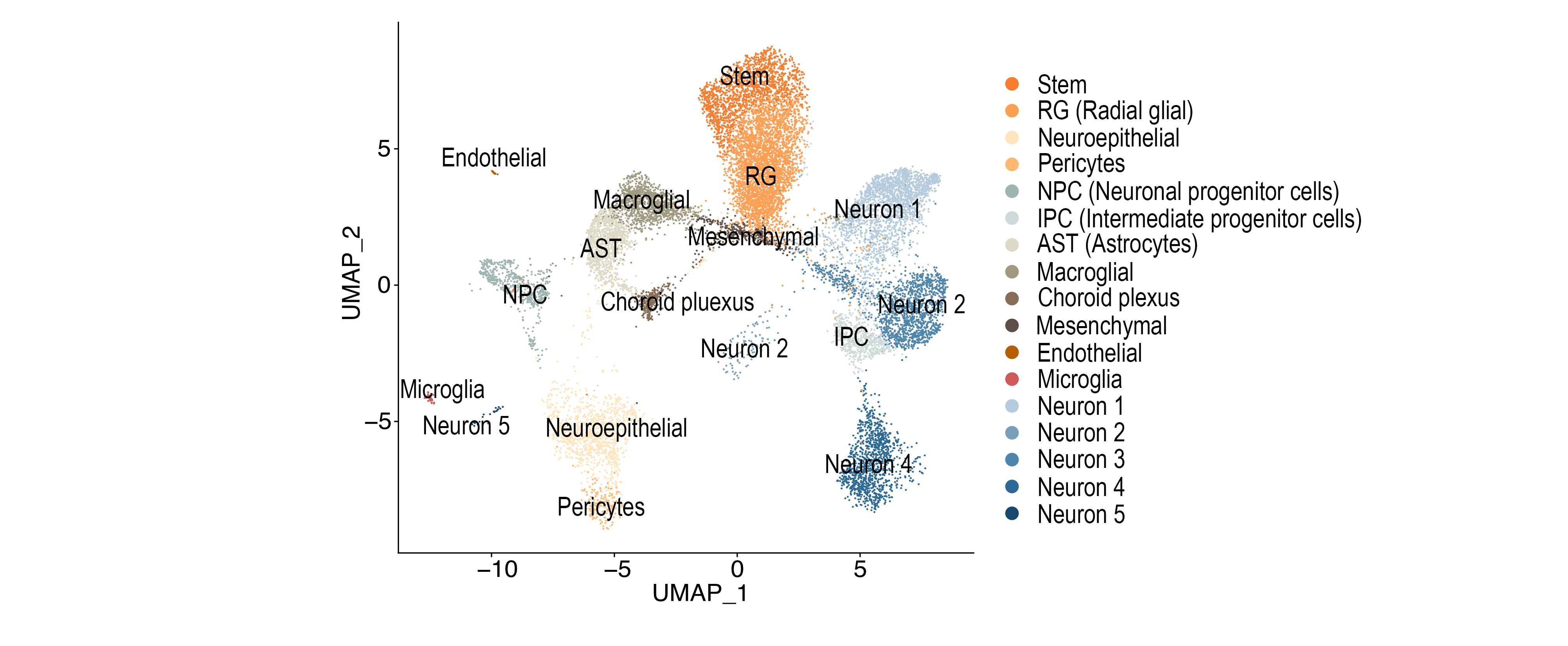

Supplement: Supplementary file 3 — Supplementary Material 3 [file 13287_2024_3732_MOESM3_ESM.png]

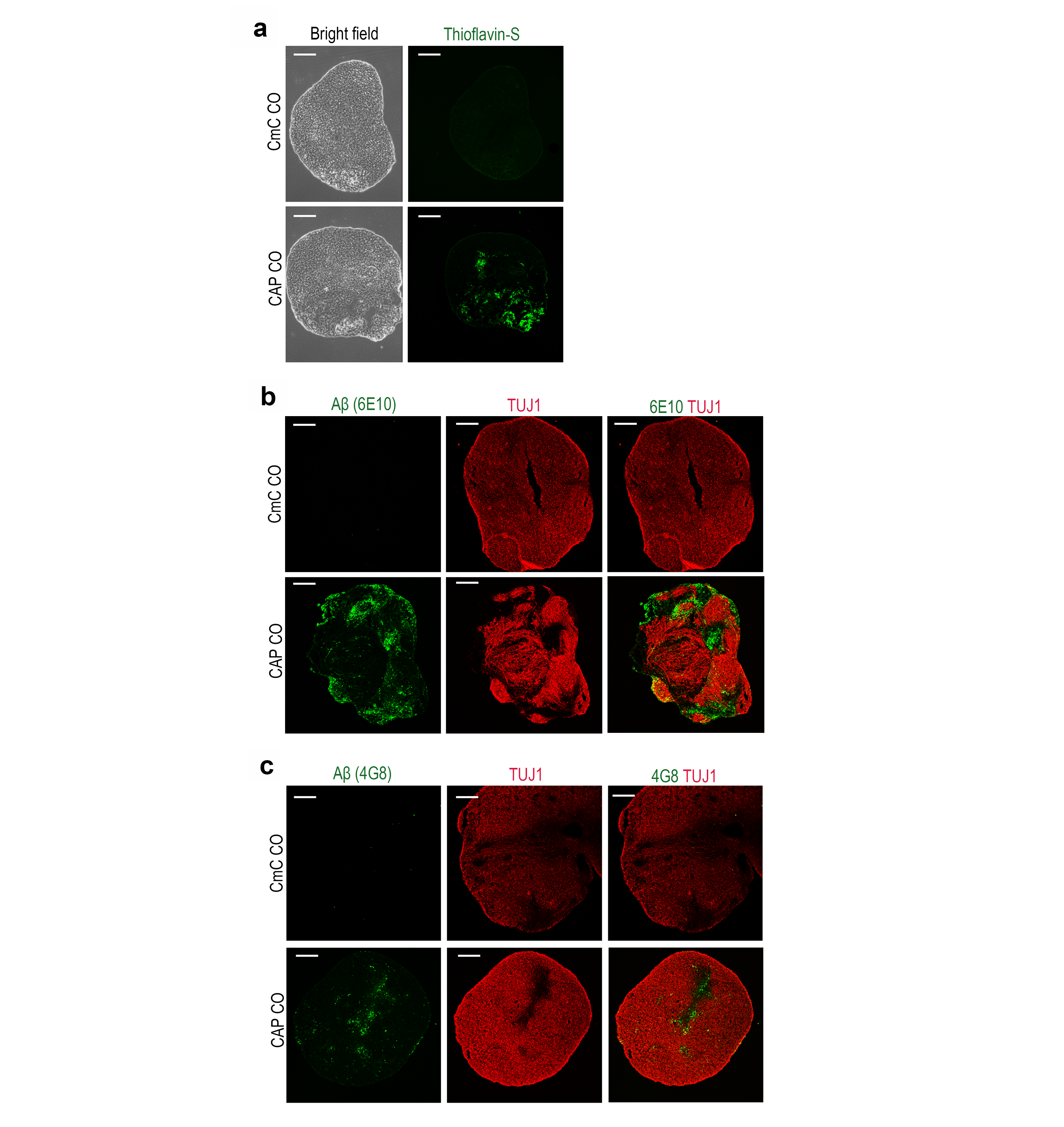

Supplement: Supplementary file 4 — Supplementary Material 4 [file 13287_2024_3732_MOESM4_ESM.png]

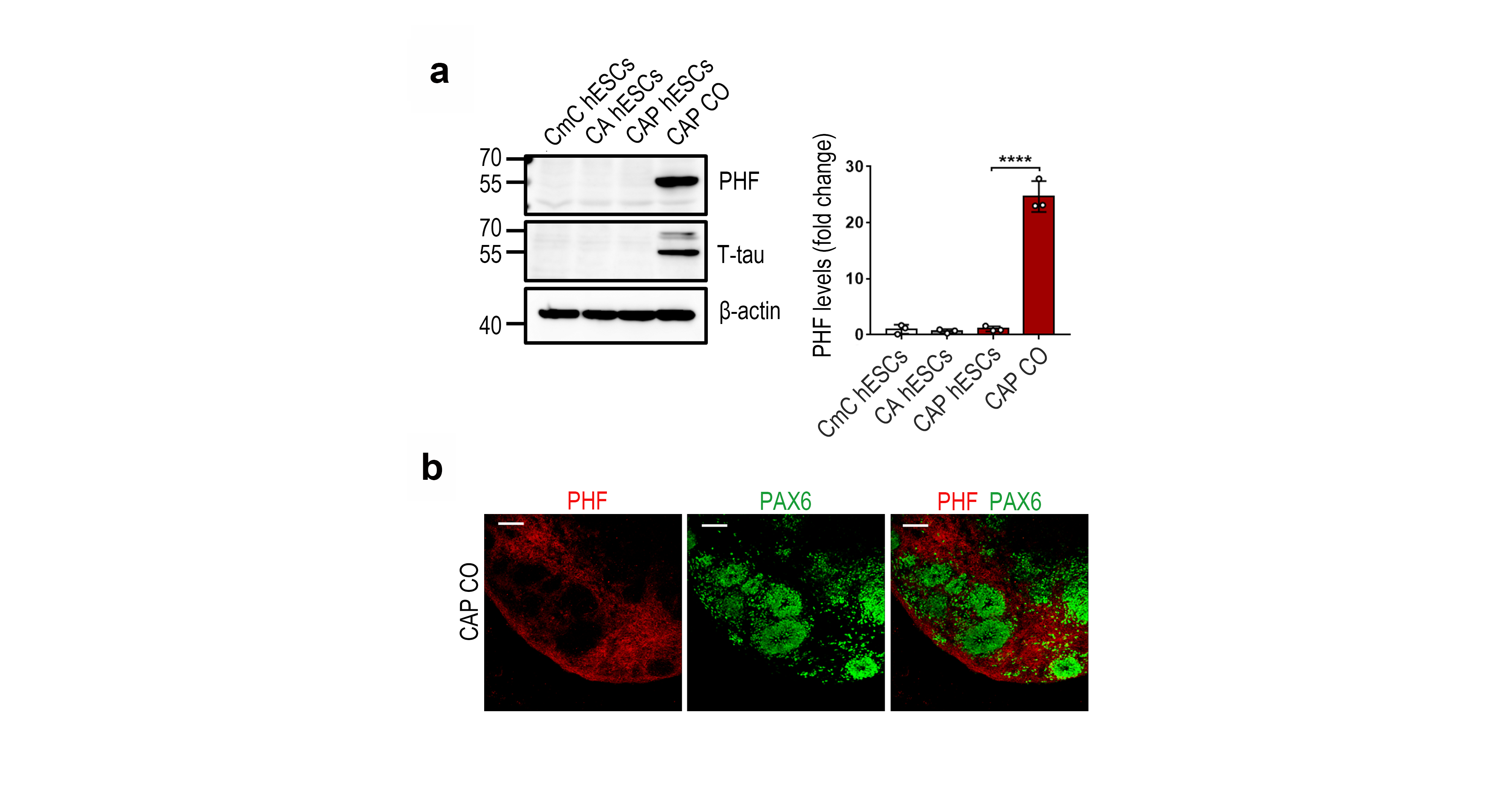

Supplement: Supplementary file 5 — Supplementary Material 5 [file 13287_2024_3732_MOESM5_ESM.png]

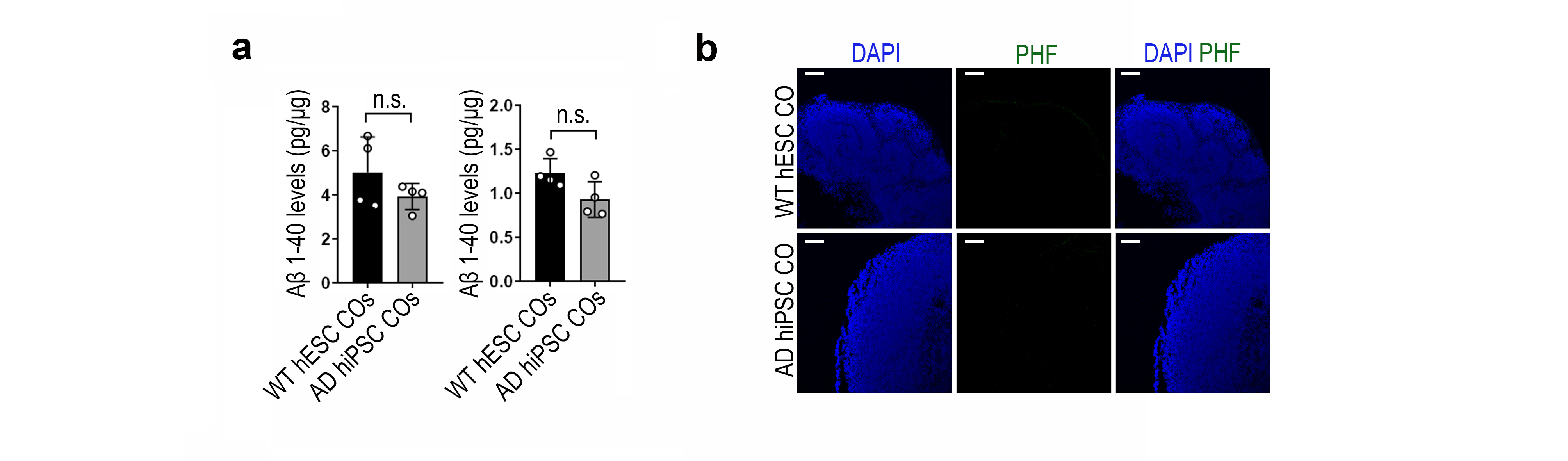

Supplement: Supplementary file 6 — Supplementary Material 6 [file 13287_2024_3732_MOESM6_ESM.png]

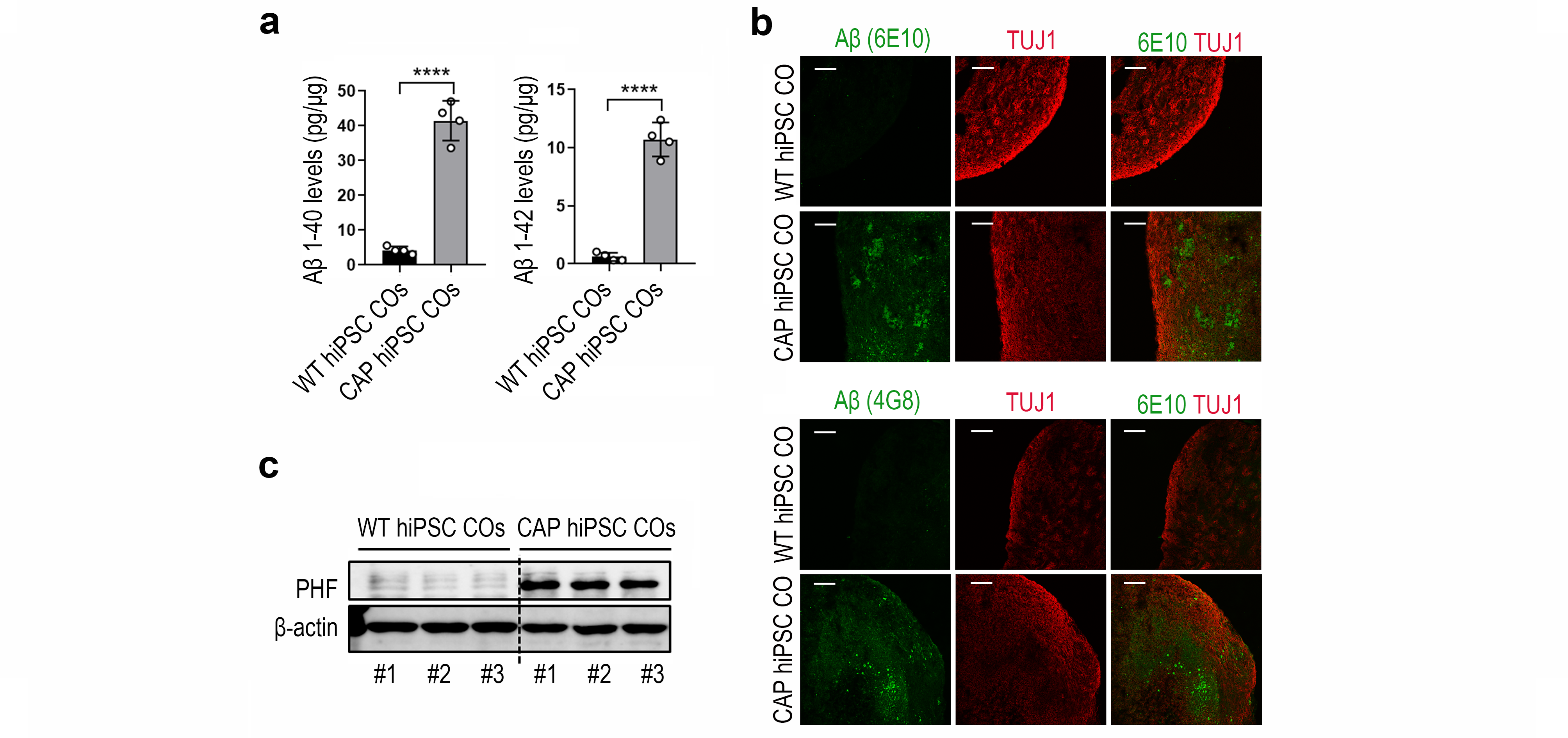

Supplement: Supplementary file 7 — Supplementary Material 7 [file 13287_2024_3732_MOESM7_ESM.png]

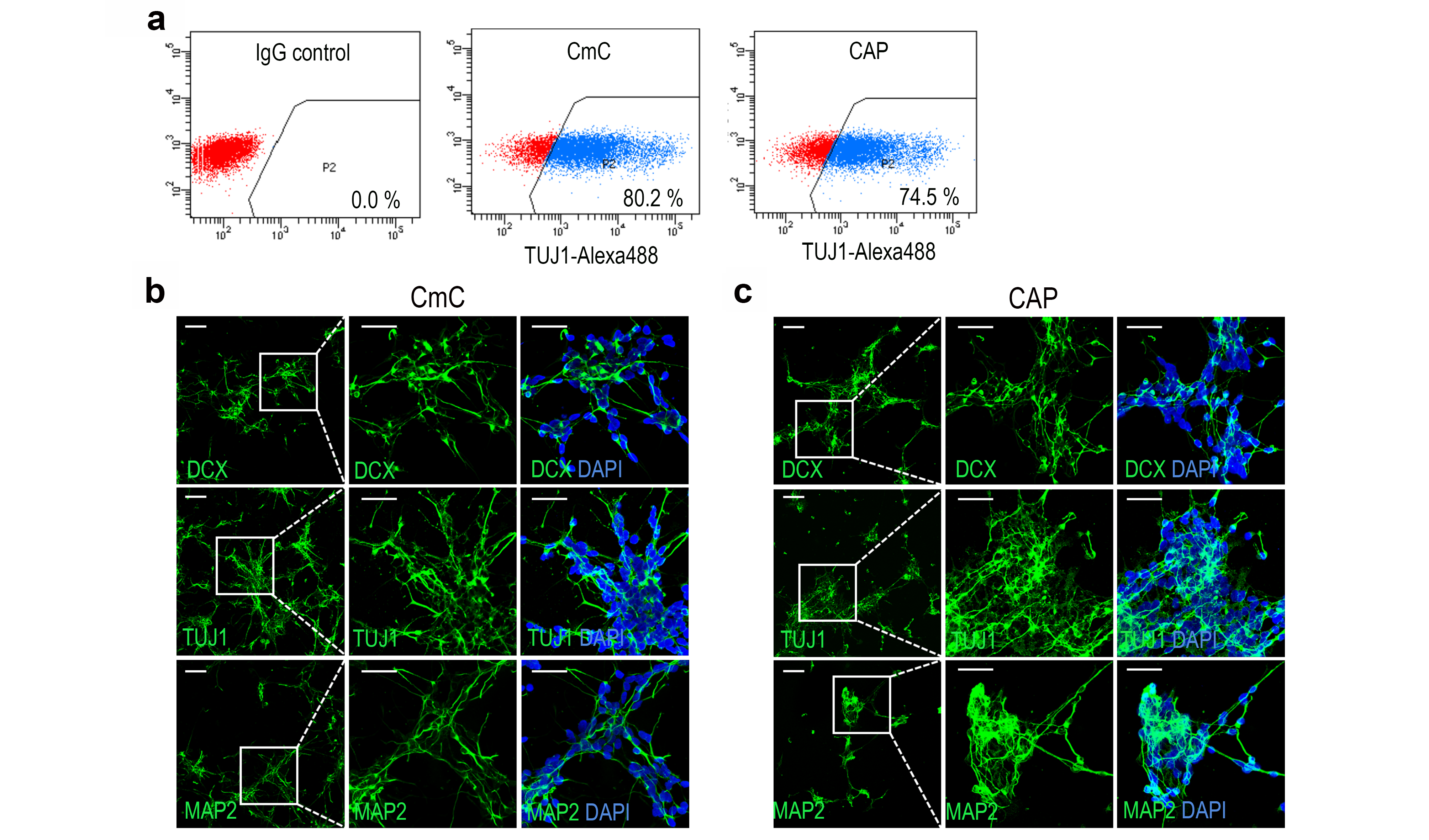

Supplement: Supplementary file 8 — Supplementary Material 8 [file 13287_2024_3732_MOESM8_ESM.png]

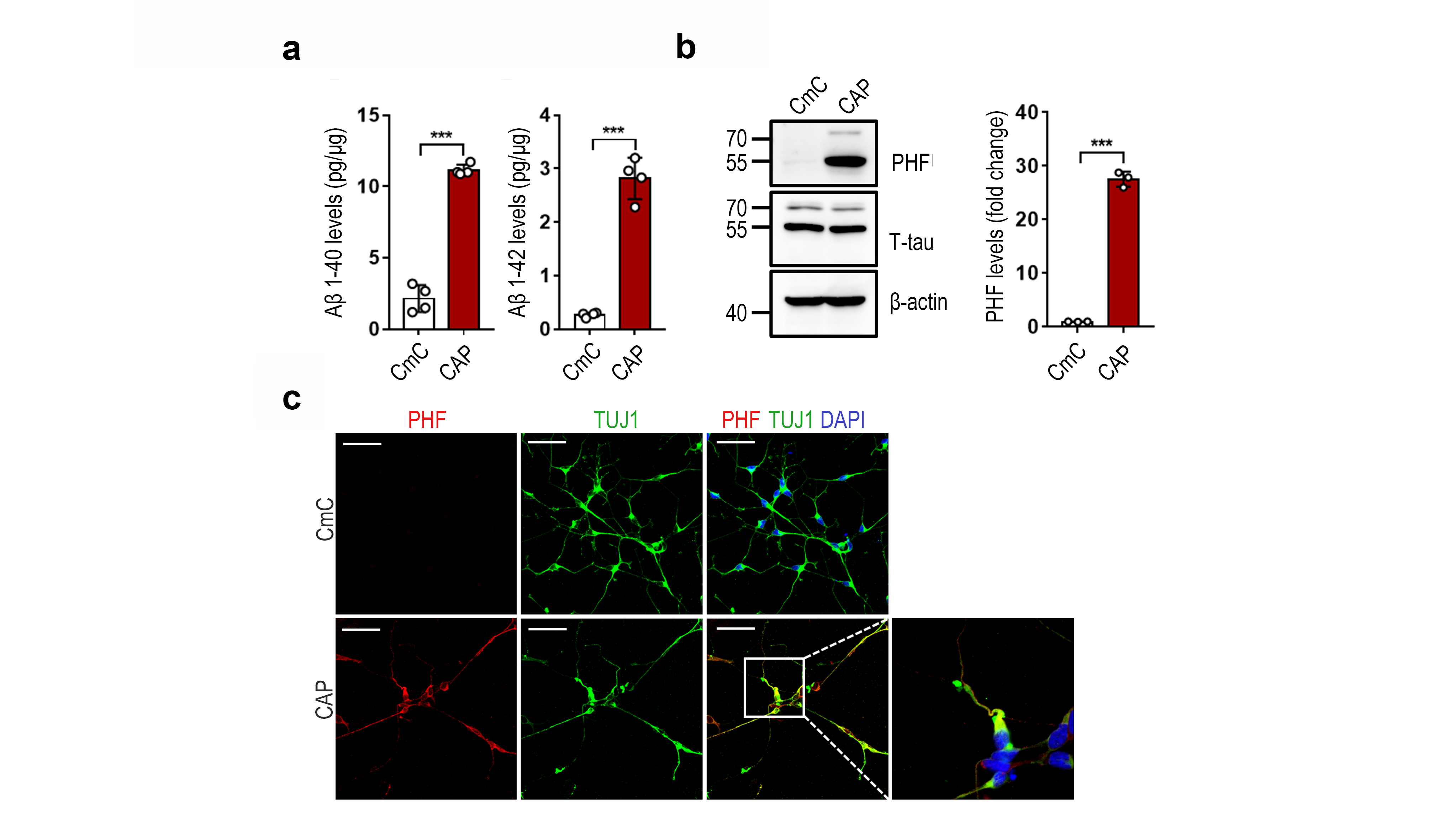

Supplement: Supplementary file 9 — Supplementary Material 9 [file 13287_2024_3732_MOESM9_ESM.png]

Fig. 1B

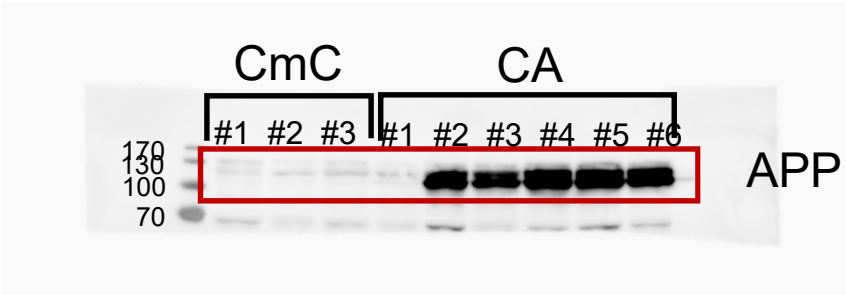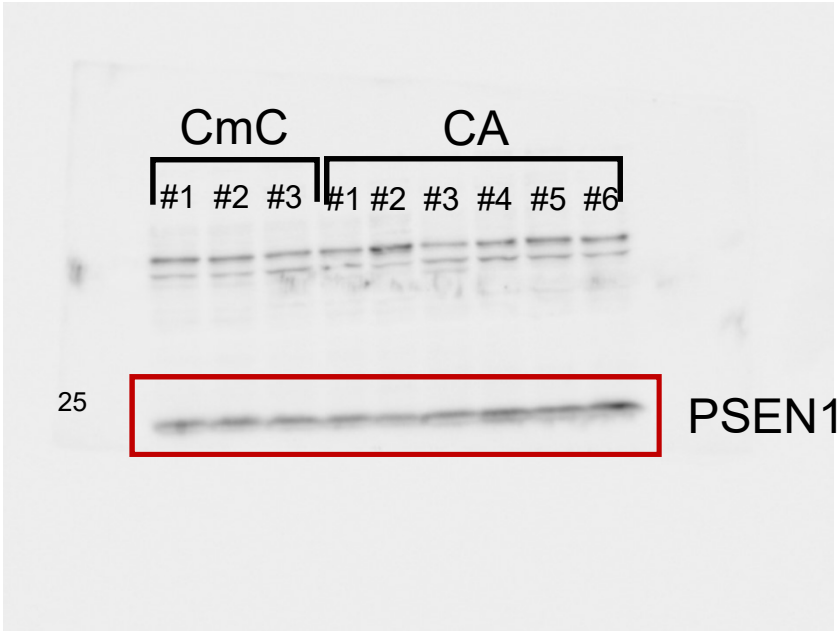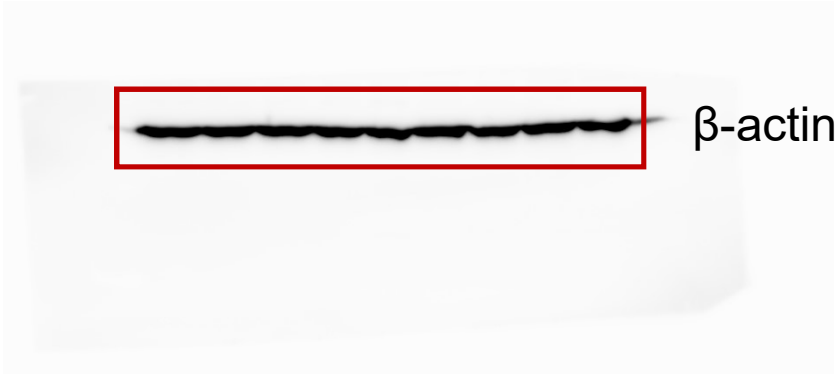

Fig. 1B

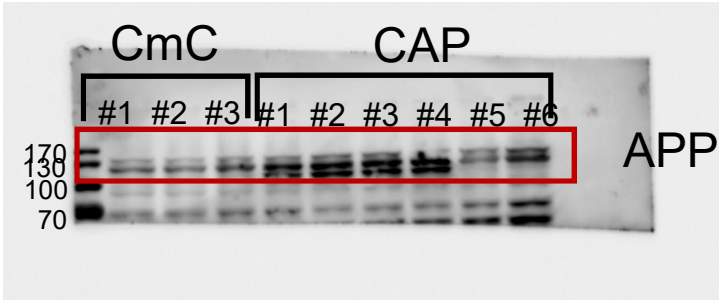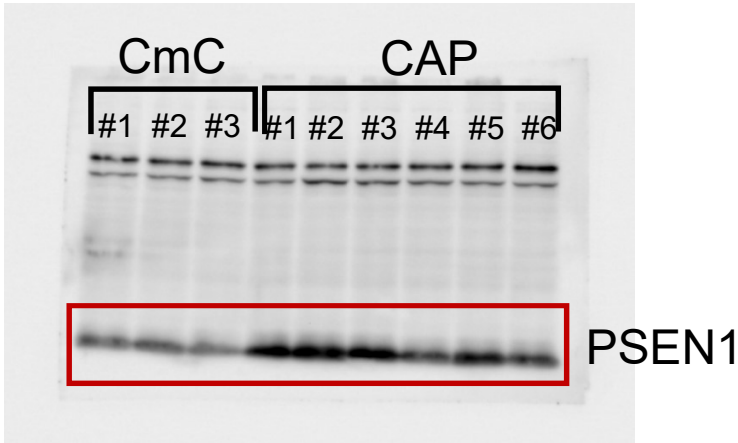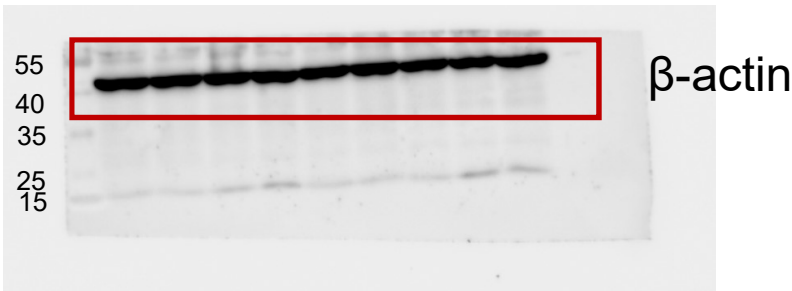

Fig. 3C

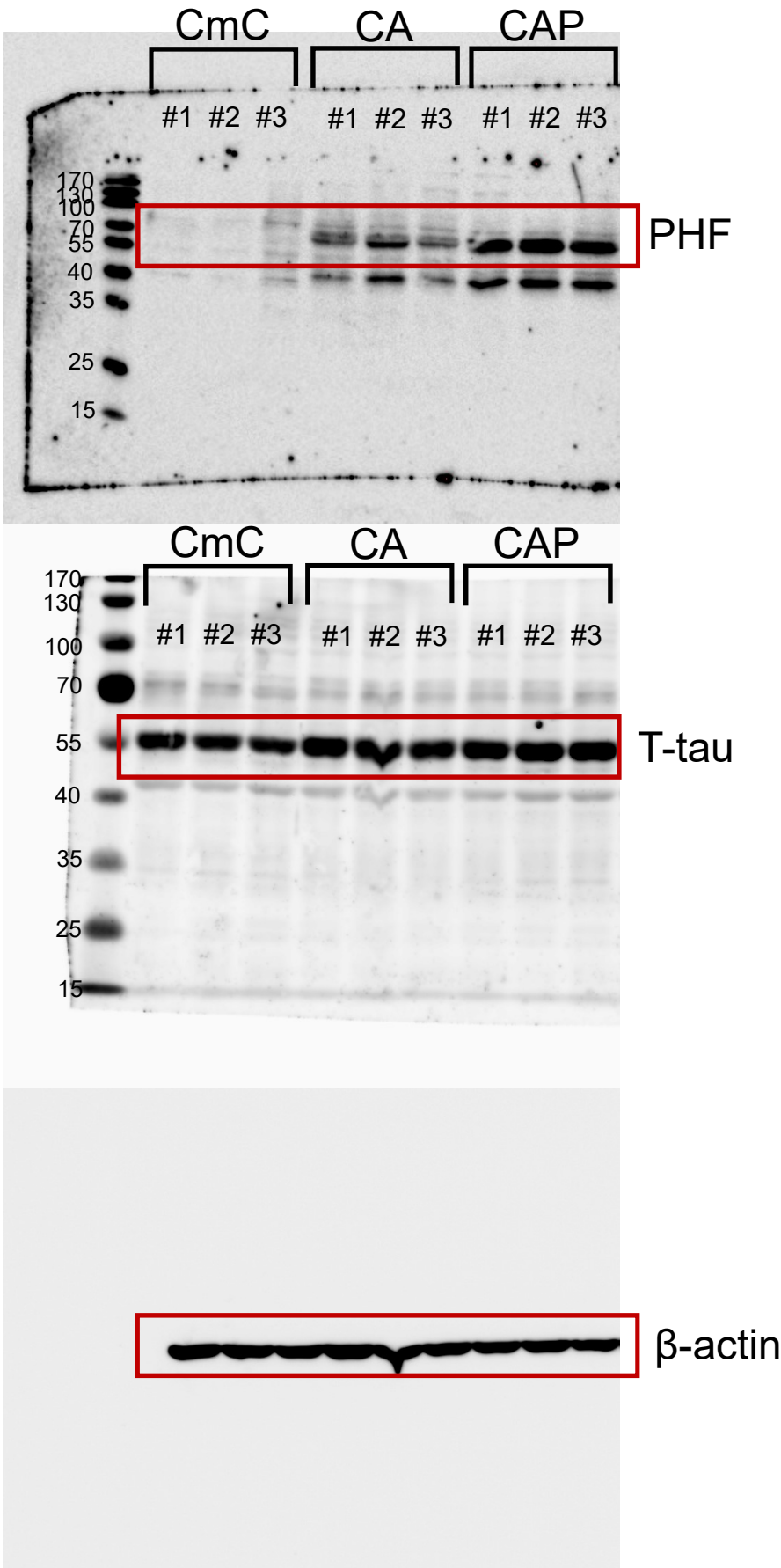

Fig. 4C

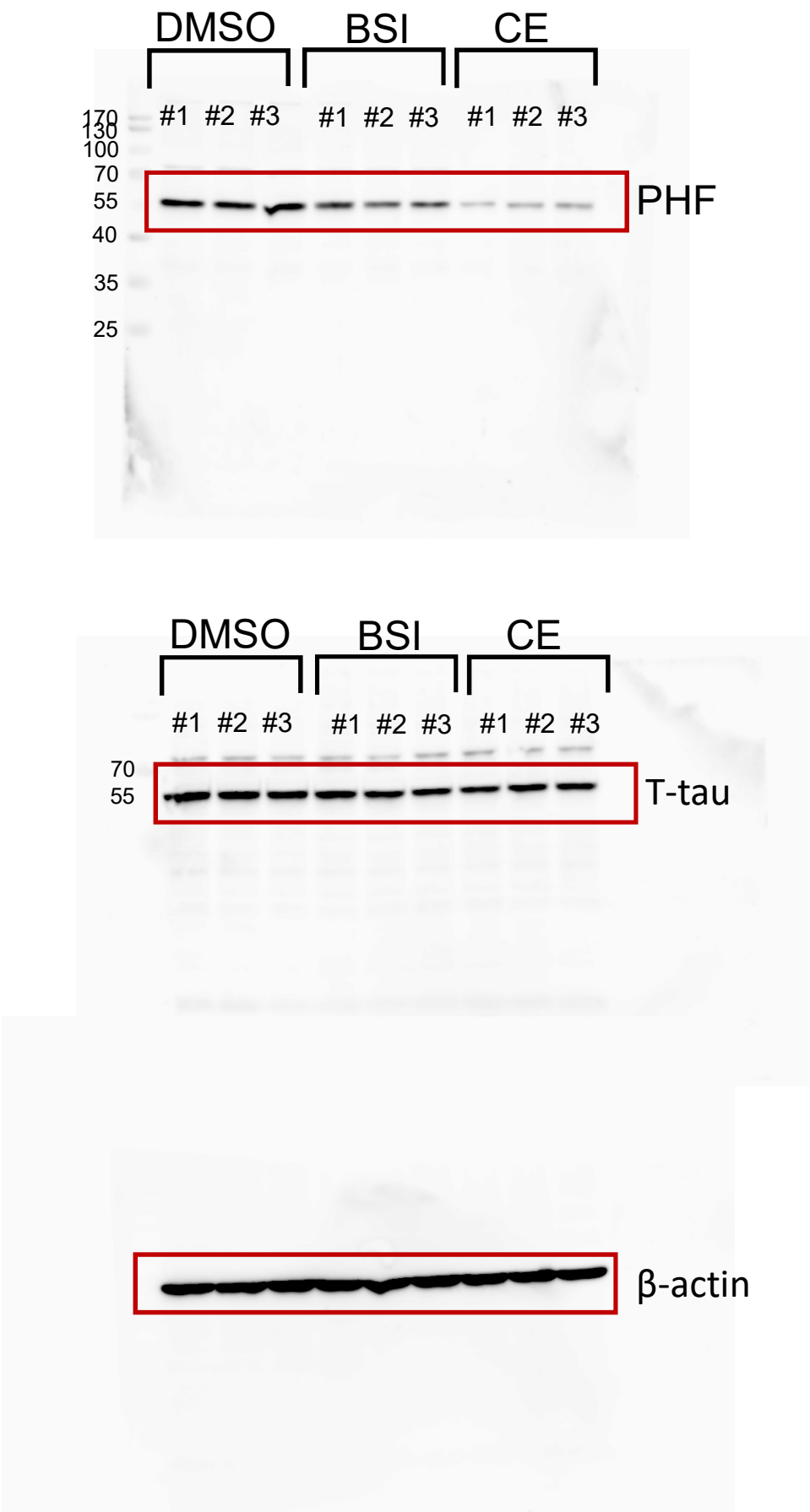

Fig. 6C

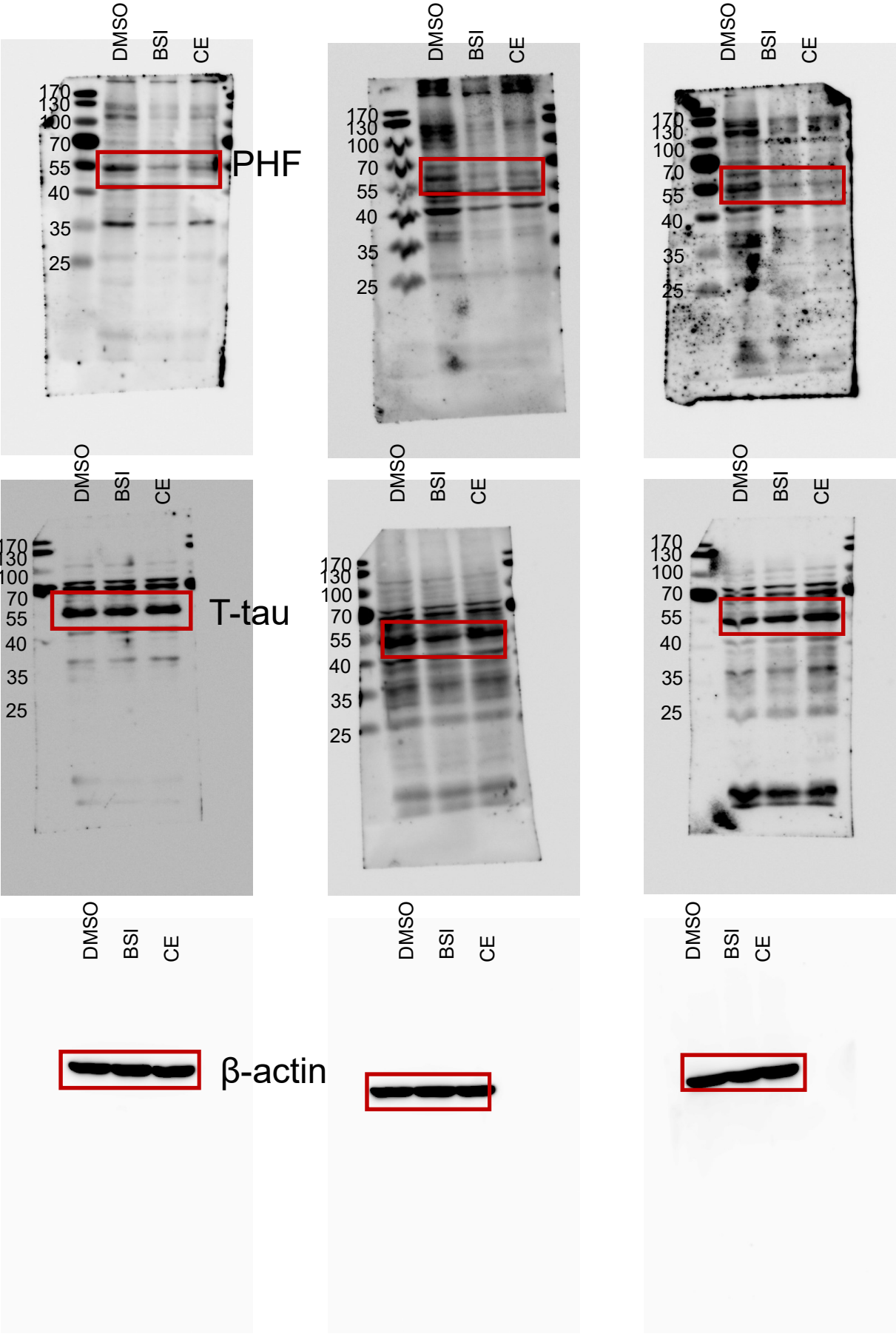

Supplementary Fig. 3A

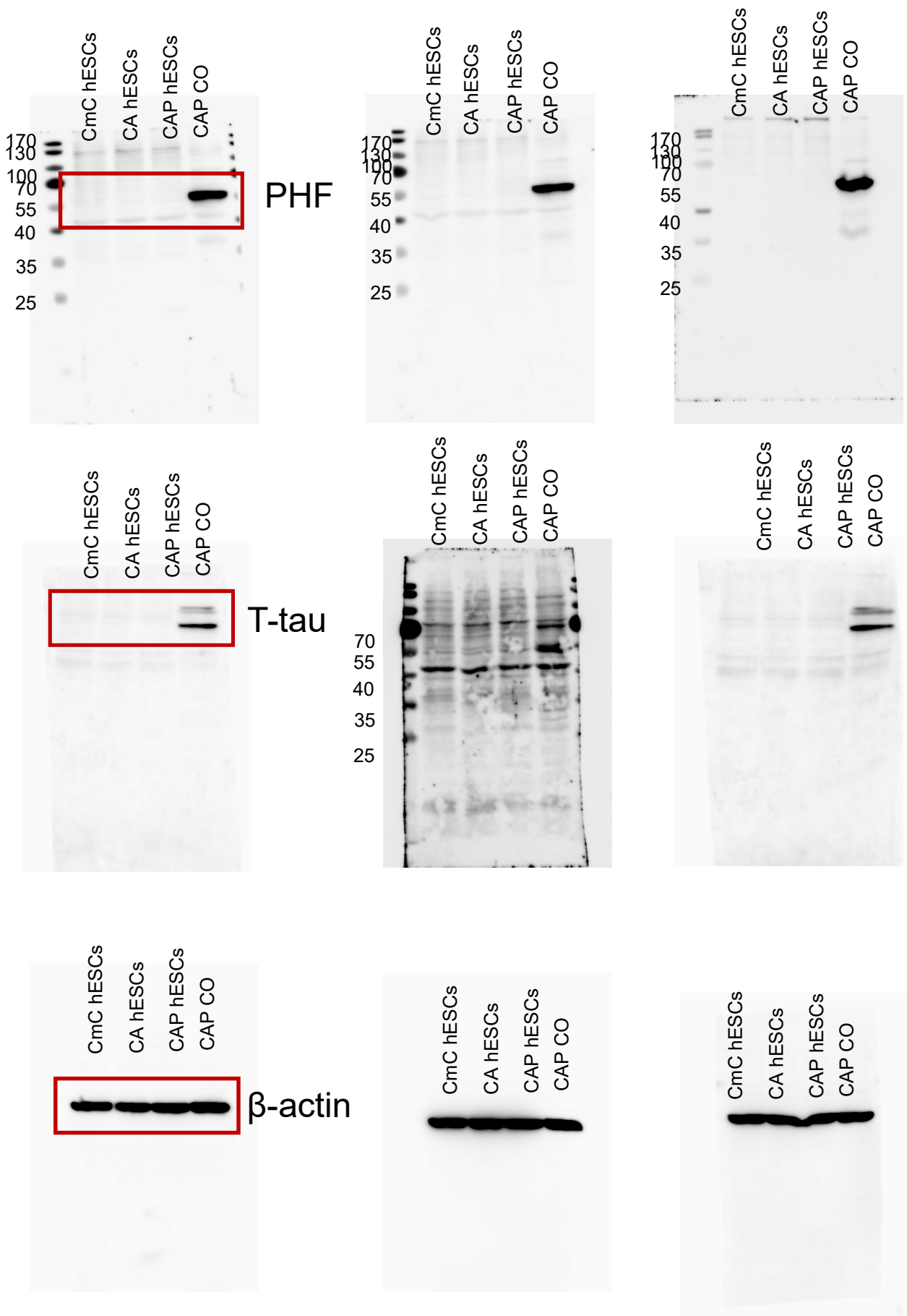

Supplementary Fig. 5B

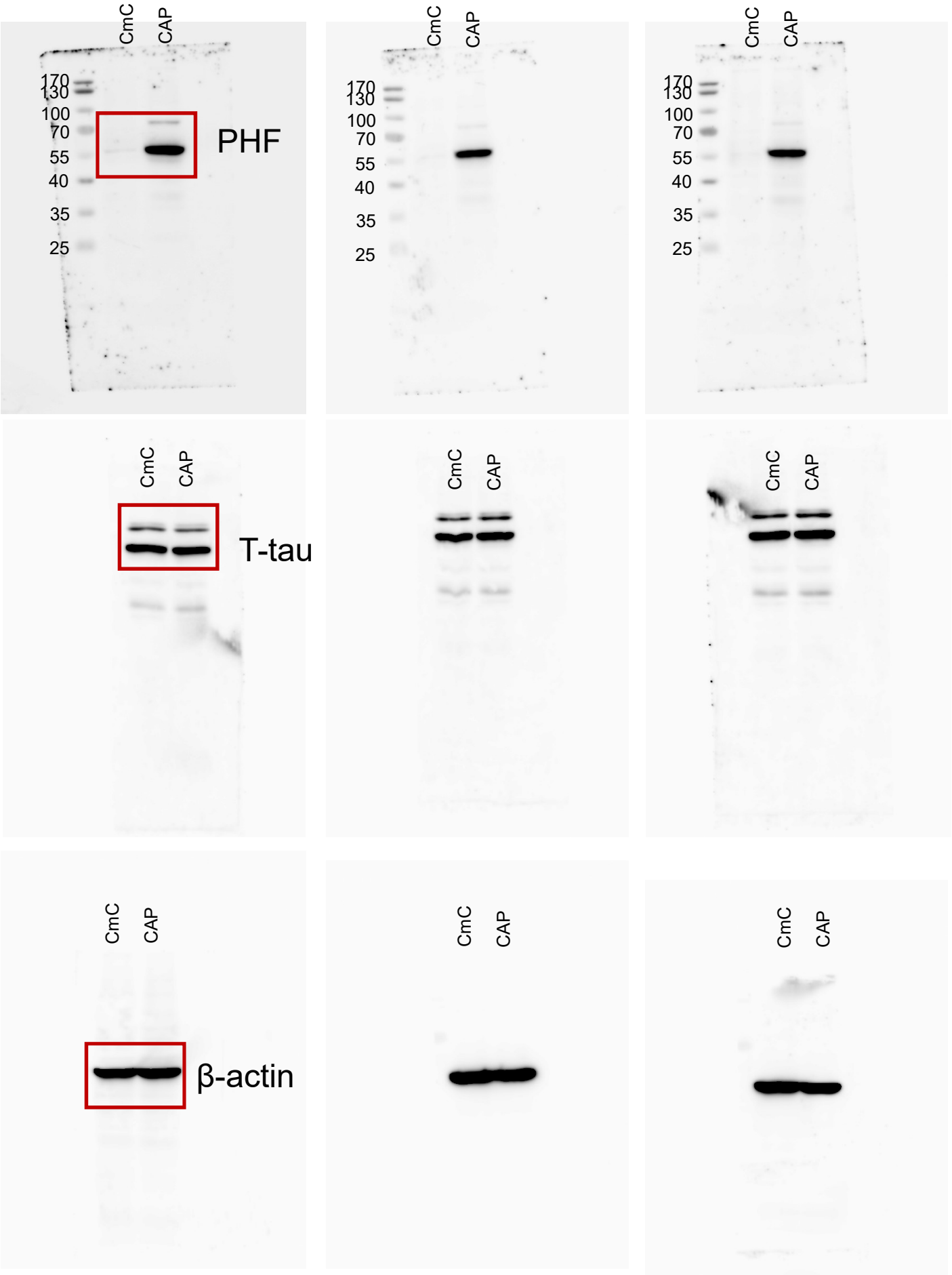

Supplement: Supplementary file 10 — Supplementary Material 10 [file 13287_2024_3732_MOESM10_ESM.pdf]
