# Supplementary material for: Simple modeling of familial Alzheimer’s disease using human pluripotent stem cell-derived cerebral organoid technology
Source: Stem Cell Res Ther. 2024 Apr 24;15:118. doi: 10.1186/s13287-024-03732-1 (PMC11040922; doi:10.1186/s13287-024-03732-1)
Supplement: Supplementary file 11 — Supplementary Material 11 [file 13287_2024_3732_MOESM11_ESM.docx]

**Supplementary information**

**Simple modeling of familial Alzheimer’s disease using human pluripotent stem cell-derived cerebral organoid technology**

Mu Seog Choe^a,1^, Han Cheol Yeo^a,1^, Joong Sun Kim^b,1^, Jean Lee^c^, Hae Jun Lee^d^, Hyung-Ryong Kim^e^, Kyung Min Baek^f^, Na-Yeon Jung^g^, Murim Choi^c,^*, Min Young Lee^a,^*

**Figure legends**

**Fig. S1** **Selection of the optimal promoter for stable gene expression of familial Alzheimer’s disease (fAD).** (a) Schematic representation of the procedures for the establishment of human pluripotent stem cell (hPSC) lines and the generation of cerebral organoids (COs). (b) Representative fluorescence microscopy images of the established CmC, PmC, EmC, and CMmC human embryonic stem cell (hESC) lines. Scale bar = 200 μm. (c) The maintenance period of mCherry expression during the CO formation. Representative fluorescence microscopy images taken were at the indicated time points. Scale bar = 500 μm. Abbreviations: CAG, CMV early enhancer/chicken β-actin; PGK, phosphoglycerate kinase; EF1α, elongation factor 1α; CMV, cytomegalovirus

***Fig. S2 Localization of the CAP lentiviral vector integration site.*** *(a) Plasmid map of the CAP lentiviral vector and the position of the integration site on human chromosome 17. (b) Sequence of chimeric reads that harbor both the CAP vector and chromosome 17 sequences aligned to the integration site. CAP vector sequences were covered at a mean depth of 12×, which suggests a single copy insertion.*

***Fig. S3. Single cell-level clustering of a 70-day-old CAP cerebral organoid (CO).*** *UMAP: uniform manifold approximation and projection*

**Fig. S4 Increase of amyloid-β (Aβ) in an Alzheimer’s disease (AD) cerebral organoid (CO) model.** COs were subjected to analysis with thioflavin-S staining and immunohistochemistry at day 70. (a) Detection of Aβ in the CmC and CAP COs with thioflavin-S staining. Scale bar = 200 μm. (b, c) Representative immunohistochemistry images of Aβ in the CmC and CAP COs. Scale bar = 200 μm.

**Fig. S5** **Expression of paired helical filament (PHF) tau in the neuronal cells of AD cerebral organoids (COs).** COs were analyzed by western blotting and immunohistochemistry staining at day 70. (a) PHF-tau and total tau levels in CmC, CA, and CAP human embryonic stem cells (hESCs), and CAP COs were analyzed by western blot analysis from the lysates of each sample. The data represents the mean ± standard error of the mean (SEM); *****P* < 0.0001 vs. CAP hESCs; n = 3 per sample. (b) Representative immunohistochemistry images showing the PHF-tau and PAX6 levels in the CAP CO. Scale bar = 100 μm.

***Fig. S6 Comparison of amyloid-β (Aβ) and paired helical filament (PHF)-tau levels between cerebral organoids (COs) generated from wild-type human embryonic stem cell cerebral organoids (WT hESC CO) and AD patient-derived human-induced pluripotent stem cells (AD hiPSC CO).*** *COs were subjected to ELISA and immunostaining analysis at day 70. (a) Aβ1-40 and Aβ1-42 levels in the lysates of WT hESC COs and AD hiPSC COs. The data represents the mean ± standard error of the mean (SEM); n.s. = not significant; n = 4 per sample. (b) Representative immunohistochemistry images showing the PHF-tau levels in the WT hESC COs and AD hiPSC COs. Scale bar = 100 μm.*

***Fig. S7 Increase in the amyloid-β (Aβ) and paired helical filament (PHF)-tau levels in an Alzheimer’s disease (AD) cerebral organoid (CO) model generated from human-induced pluripotent stem cells (hiPSC AD COs).*** *HiPSC-derived COs were analyzed by ELISA, western blotting, and immunostaining at day 70. (a) Aβ1-40 and Aβ1-42 levels in the lysates of wild-type and CAP hiPSC COs. The data represents the mean ± standard error of the mean (SEM); ****P < 0.0001 vs. wild-type hiPSC COs; n = 4 per sample. (b) Representative immunohistochemistry images of Aβ in the wild-type and CAP hiPSC COs. Scale bar = 100 μm. (c) PHF-tau levels in the wild-type and CAP hiPSC COs were analyzed by western blot from the lysates of each CO.*

**Fig. S8 Neuronal differentiation of Alzheimer’s disease (AD) human embryonic stem cells (hESCs).** Neuronal differentiation was induced in the CmC and CAP hESCs, and the differentiated cells were analyzed on day 50. (a) The percentage of TUJ1-positive cells was analyzed using flow cytometry. The numbers represent the percentage of TUJ1-positive cells within the indicated region. (b) Representative immunocytochemistry images showing DCX, TUJ, and MAP2 in the differentiated neuronal cells from the CmC and CAP hESCs. Scale bar = 100 μm.

**Fig. S9 Increase in the amyloid-β (Aβ) and paired helical filament (PHF)-tau levels in the Alzheimer’s disease (AD) neurons.** Neuronal differentiation was induced from the CmC and CAP human embryonic stem cells (hESC) and the differentiated cells were analyzed on day 50. (a) The levels of Aβ1-40 and Aβ1-42 in the lysates of CmC and CAP neurons. The data represents the mean ± standard error of the mean (SEM); ****P* < 0.001 vs. CmC; n = 4 per sample. (b) The PHF-tau and total tau levels in the CmC, CA, and CAP neurons were analyzed by western blot from the lysates of each sample. The data represents the mean ± SEM; ****P* < 0.001 vs. CmC; n = 3 per sample. (c) Representative immunohistochemistry images showing the PHF-tau and TUJ1 levels in the CmC and CAP COs. Scale bar = 100 μm.
